# Validation of CBCL depression scores of adolescents in three independent datasets

**DOI:** 10.1002/jcv2.12298

**Published:** 2025-01-29

**Authors:** Marie Zelenina, Daniel S. Pine, Argyris Stringaris, Dylan M. Nielson

**Affiliations:** ^1^ Section on Development and Affective Neuroscience NIMH NIH Bethesda MD USA; ^2^ Faculdade de Ciências da Universidade de Lisboa Instituto de Biofísica e Engenharia Biomédica Campo Grande Portugal; ^3^ Division of Psychiatry University College London London UK; ^4^ 1st Department of Psychiatry National and Kapodistrian University of Athens Aigineition Hospital Athens Greece; ^5^ Machine Learning Team NIMH NIH 10 Center Dr Bethesda MD USA

**Keywords:** adolescent brain cognitive development, CBCL, depression, informant discrepancy, measure validation

## Abstract

**Background:**

Depression is common, burdensome, and is frequently first diagnosed in adolescents. The popular Adolescent Brain Cognitive Development dataset (ABCD) provides an attractive opportunity to research depression in adolescents. The only continuous measure of depression, as defined by DSM‐5, in ABCD is the Child Behavior Checklist's DSM‐5‐Oriented Affective Problems scale (CBCL‐Aff). We validated CBCL‐Aff in the ABCD data and confirmed our results on two independent datasets: the Healthy Brain Network (HBN) and the Brazilian High Risk Cohort Study (BHRC).

**Methods:**

We tested Sensitivity, Specificity and Strict Specificity. Our participants were aged 8–11 years (ABCD) or 8–12 years (HBN, BHRC). Sample size was 183–1189 participants, depending on the analysis. Sample sizes and positive case ratios were established from power estimations. We evaluated goodness of prediction with AUCROCs.

**Results:**

In ABCD with parent‐report diagnoses, CBCL‐Aff had the AUCROC value of 0.95 in the Sensitivity and 0.87 in the Specificity analysis. In ABCD with child‐report diagnoses, CBCL‐Aff had the AUCROC of 0.62 (Sensitivity), 0.48 (Specificity) and 0.46 (Strict Specificity). In HBN and BHRC, CBCL‐Aff successfully predicted clinician‐report diagnoses (HBN Sensitivity: AUCROC = 0.86, Specificity: AUCROC = 0.71; BHRC Sensitivity: AUCROC = 0.90, Specificity: AUCROC = 0.80, Strict Specificity: AUCROC = 0.78).

**Conclusions:**

We validated CBCL‐Aff as a measure of depression in adolescents aged 8–11 years and we recommend its use with the following limitation: as parents and children disagreed on the child's symptoms, we discuss implications of using a parent‐report only measure of child depression.

## Graphical abstract

Depression is common, burdensome, and is frequently first diagnosed in adolescents, thus the popular Adolescent Brain Cognitive Development dataset (ABCD) provides an attractive opportunity to research depression in adolescents. The only DSM‐5‐oriented continuous measure of depression in ABCD is the parent‐report Child Behavior Checklist's DSM‐5‐Oriented Affective Problems scale (CBCL‐Aff). We validated the CBCL‐Aff in the ABCD dataset, and verified our findings on two independent datasets which provided clinicians' diagnoses: the Healthy Brain Network (HBN) and the Brazilian High Risk Cohort Study (BHRC).

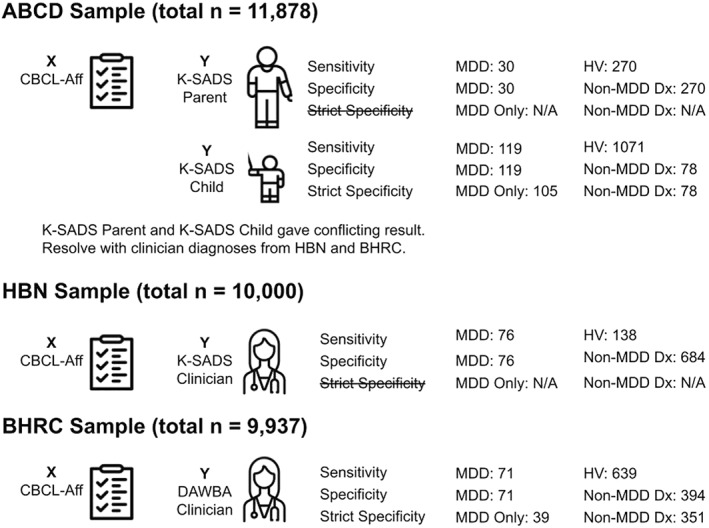

**Key points**

**What's known?**

- CBCL‐Aff is the only continuous measure of depression in the highly popular ABCD dataset, as well as in other studies.

**What's relevant?**

- Using valid measures is important to ensure the quality of research that uses them, and they are essential for measurement‐based care.

**What's new?**

- We validated CBCL‐Aff as a continuous measure of depression in children aged 8–11 in the ABCD dataset and confirmed our finding on two independent datasets which had independent clinician's diagnoses: HBN and BHRC.
- CBCL‐Aff correlates well with parent report symptoms and clinician diagnosis, but not with child self‐report of symptoms.
- We found CBCL‐Aff to be valid but researchers should keep in mind that it is not necessarily reflective of the children's view of themselves.

## INTRODUCTION

Depression (major depressive disorder, MDD) is common (WHO, 2021) and potentially lethal (Nasser & Overholser, 1999; Turecki & Brent, 2016). Depression research in adolescents is particularly important because depression originates in early life (Costello et al., 2003). The Adolescent Brain Cognitive Development study (ABCD) (Casey et al., 2018) is a longitudinal project that collects multimodal data from adolescence (8–10 years old) to early adulthood (20 years old). It is the largest long‐term study of child brain development and health in the United States (Karcher & Barch, 2021). ABCD provides data on childhood experiences, biology, as well as behavioral and other outcomes. The study has recently released data covering nine data collection waves over 4 years, with participants currently being between 13 and 14 years old. Many investigators have studied depression in the ABCD data (Ho et al., 2022; Klein et al., 2022; Lee et al., 2022; Liu et al., 2022; Xiang et al., 2022) and as more data is released from adolescent years, this will only accelerate.

While ABCD's scope makes it a tantalizing resource for adolescent depression research, it has few measures of depression. Dichotomous diagnostic information is derived from the lay‐administered computerized Kiddie Schedule for Affective Disorders and Schizophrenia (KSADS‐COMP) (Townsend et al., 2020). As a dichotomous variable, the KSADS diagnoses are simpler and more interpretable than continuous measures but reduce statistical power due to information loss. Additionally, the use of dichotomous diagnoses increases the risk of false negatives, because the change in one criterion can eliminate the diagnosis, as well as the risk of false positives (Altman & Royston, 2006). Nevertheless, problems exist with structured psychiatric interviews that are administered by lay interviewers due to their lack of clinical experience. These problems raise questions about reliability and validity in various contexts (Townsend et al., 2020). Moreover, previous releases of the ABCD data had errors in diagnostic codes generated from the K‐SADS interviews (Barch et al., 2021); however, these problems were addressed in the latest data release 5.1 which we are using in this work. Given these and other issues, many groups searching for correlates of adolescent depression in ABCD have used one of the Child Behavior Checklist (CBCL; Achenbach & Edelbrock, 1991a) depression subscales (Ho et al., 2022; Lee et al., 2022; Xiang et al., 2022). These depression subscales are the ABCD's only continuous measures of depression severity data in early waves, joined by the Youth Self‐Report form in mid‐Year 4 (Barch et al., 2021). Thus, confirming that the CBCL's depression subscales are valid in the ABCD sample will support the validity of past and future work on adolescent depression in ABCD (Flake & Fried, 2020; Fried et al., 2022).

Of course, the CBCL has been validated in previous studies (Kazdin et al., 1983; Leung et al., 2006). The CBCL has three depression subscales, two empirically derived from factor analysis (Withdrawn‐Depressed and Anxious‐Depressed), and one based on the DSM‐5 criteria (Affective Problems). These scales have generally been validated (Clarke et al., 1992; Ebesutani et al., 2010; Ferdinand, 2008; Lacalle et al., 2012; Nakamura et al., 2009; Skarphedinsson et al., 2021), but the validation focused mainly on clinically referred samples (Ebesutani et al., 2010; Ferdinand, 2008; Lacalle et al., 2012; Nakamura et al., 2009; Skarphedinsson et al., 2021), while the sample in the ABCD dataset is collected to be representative of the general US population. Moreover, the age range in all these validation studies (Ebesutani et al.: 6.55–18.9 years; Nakamura et al.: 4.2–19.7 years; Skarphedinsson et al.: 6.1–17.8 years; Lacalle et al.: 8–17 years) is wider than the age range of children in the baseline wave of the ABCD dataset (8–10 years).

The CBCL is a parent‐report measure. Previous literature finds discrepancies between parent‐report and child‐report measures of depression (Abate et al., 2018; Angold et al., 1987; Baumgartner et al., 2020; Kashani et al., 1985) and warns against using parent‐report measures as a proxy for children's affective state (Abate et al., 2018). Previous research on CBCL‐AFF validation noticed low to moderate agreement between parents and children (Lacalle et al., 2012). Moreover, many items in the CBCL's depression scales ask about internal mental states that parents may not see or know. Thus, we hypothesized that CBCL's depression subscales would not be valid measures of depression in children.

While our primary concern is the validity of the CBCL in the ABCD sample, the ABCD does not provide diagnostic information from an independent clinician so we have no way to adjudicate between parent report and child report information. Therefore, we had to expand our analyses to two additional large studies of adolescent mental health: the Brazilian High Risk Cohort (BHRC; Salum et al., 2015) and the Healthy Brain Network (HBN; Alexander et al., 2017). Both studies provide clinician consensus diagnoses. HBN and BHRC both represent large‐scale data collection efforts with 10,000 (HBN) or 9937 (BHRC) participants in New York, USA (HBN) and Porto Alegre and Sao Paolo, Brazil (BHRC), respectively. HBN used a community‐referred recruitment model, specifically focusing on families with concerns about their child's mental health. BHRC used screening of the families to identify a generally representative sample and a sample of children with high risk of having a mental health disorder.

**FIGURE 1:**
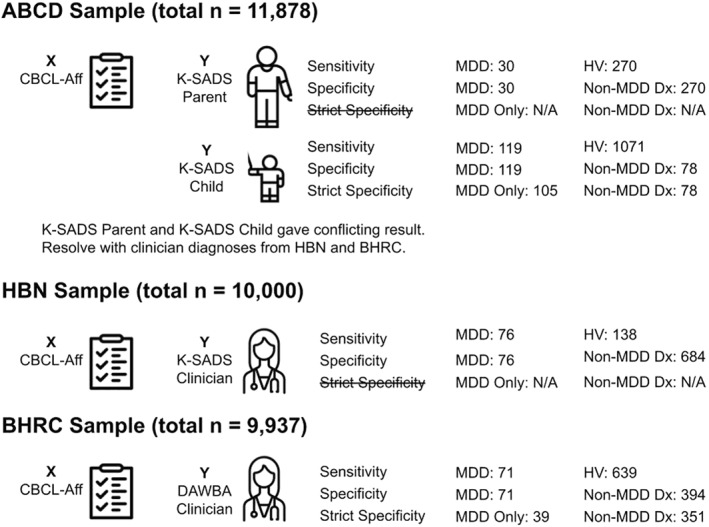
Sample sizes for all samples (ABCD, HBN, BHRC) and all analyses (Sensitivity, Specificity, Strict Specificity). ABCD, Adolescent Brain Cognitive Development; BHRC, Brazilian High Risk Cohort study; CBCL‐Aff, the Child Behavior Checklist's DSM‐5‐Oriented Affective Problems scale; DAWBA, the Development and Well‐being Assessment; Dx, Diagnosis; HBN, Healthy Brain Network; HV, Healthy Volunteer; K‐SADS, the Kiddie Schedule for Affective Disorders and Schizophrenia; MDD, Depression/Major Depressive Disorder; NA, Not Available.

In this study we assess the validity of the CBCL's depression subscales by examining the sensitivity and specificity of the CBCL to distinguish between depressed and non‐depressed adolescents in three datasets: ABCD, HBN, and BHRC. For our purposes, sensitivity is the ability of a depression scale to distinguish between depressed and non‐depressed adolescents. Specificity is the ability of a depression scale to distinguish between depression (with or without comorbidities) and other (non‐depression) diagnoses. Since comorbidities are common in depression, it is possible that a scale might appear to be specific when it is just differentiating adolescents with many symptoms from those with few symptoms. To account for this, we also tested a stricter from of specificity (termed Strict Specificity): the ability of a depression scale to distinguish between depression without comorbidities from non‐depression diagnoses. Area Under the Receiver Operator Curve (AUCROC) is our criterion for goodness of prediction with threshold of 0.8 (see Figure 1 and Table S1 in Supporting Information S1, Figures S1‐S10 in Supporting Information S1).

Our analyses are preregistered: https://osf.io/a93ts/ and https://osf.io/vw5ek/.

## MATERIALS AND METHODS

### Data

All data have been collected by the investigators of the corresponding projects (ABCD/HBN/BHRC).

The aim of the ABCD study is to identify the foundational aspects of adolescent brain development and environmental details that shape a person's future (Casey et al., 2018). We are using the data collected at baseline (age 8–11). The full sample includes 11,878 participants from 21 study sites. Enrollment details, ethical approvals, and inclusion/exclusion criteria for the ABCD dataset, as well as for the HBN and the BHRC, are in Supporting Information S2 and S3.

The **HBN** is an initiative to collect a large (10,000 participants) open resource dataset of child and adolescent (ages 5–21) brain development that includes psychiatric, behavioral, cognitive, lifestyle phenotypes, and multimodal brain imaging (resting and naturalistic viewing fMRI, diffusion MRI, morphometric MRI), electroencephalography, eye‐tracking, voice and video recordings, genetics and actigraphy (Alexander et al., 2017), with the aim to further the study of child and adolescent mental illness. The sample was recruited by seeking out parents concerned about their child's mental health/behavior (Alexander et al., 2017). As a result, the diagnostic rates in this sample are quite high.

The **BRHC** sample (*n* = 9,937, out of which 2512 were evaluated with diagnostic instruments) was designed to understand developmental trajectories of psychopathology and mental disorders in youth. The high risk sample was based on early psychiatric symptoms and high family loading of psychopathology (Salum et al., 2015).

Since neither HBN nor BHRC had enough participants in the 8–11 years old range (which would match the ABCD sample), we extended our analysis to include participants in the 8–12 year old range in these datasets.

#### Missing data

As a rule, we omitted participants with any missing K‐SADS/DAWBA diagnosis data or missing CBCL‐AFF scores, but there were some minor exceptions as described in Supporting Information S4.

### Measurements

#### Input measures in all three datasets: CBCL‐Aff, CBCL‐AD, CBCL‐WD

All three datasets (ABCD, HBN, BHRC) use the parent‐report Child Behavior Checklist (CBCL) (Achenbach & Edelbrock, 1991b) to assess behavioral and emotional problems in children and adolescents. The full CBCL for children aged 6–18 is comprised of 113 items, of which 67 are included in one of the six DSM‐oriented scales. All questions are scored on a three‐point Likert scale (0 = Not True (as far as you know), 1 = Somewhat or Sometimes True, 2 = Very True or Often True). The original CBCL scales were empirically based and grouped into higher‐order internalizing and externalizing scales (ASEBA, 2021). Items for CBCL‐Aff were selected by expert review, whereby experts rated each ASEBA item on a scale from 0 (not consistent with the DSM category) to 2 (very consistent with the category). Then, the ASEBA item was assigned to a scale if 60% experts rated the item as 2, resulting in a scale with 13 items.

We use three scales: the CBCL DSM‐oriented scale (CBCL‐Aff), which corresponds to Dysthymic (DYS) and Major Depressive Disorders (MDD) (Ebesutani et al., 2010), and the empirically based Anxious/Depressed scale (CBCL‐AD) and the Withdrawn/Depressed scale (CBCL‐WD). The list of items included in each scale can be found, Table S5 in Supporting Information S5.

#### ABCD reports: Parent‐report CBCL, child‐report BPM

Besides analyzing the parent‐report CBCL‐Aff, CBCL‐AD and the CBCL‐WD scales in the ABCD data, we also looked at the internalizing scale in the child‐report Brief Problem Monitor (BPM). BPM is not available for the baseline wave, so instead we used the wave with the most participants with depression according to the KSADS‐COMP both in parent‐report and child‐report data, which was the second‐year follow‐up wave.

#### Comparison between sex data

Because we analyzed raw CBCL values (and not t‐scores), which are not normalized by sex, and following previous research (Ebesutani et al., 2010), we initially split the participants into two groups: boys and girls (as indicated by sex assigned at birth). Because the results on boys and girls we similar (Tables S9‐1, S9‐2 in Supporting Information S9), to maximize the number of participants, we then analyzed pooled sex data.

#### Output measurements: Diagnoses

**ABCD: Parent‐reported and child‐reported K‐SADS.** The ABCD study does not provide diagnosis information based on clinician assessment. Instead, it uses the computerized version of K‐SADS: K‐SADS‐COMP (Townsend et al., 2020), completed by parents or children. K‐SADS‐COMP has been derived from “paper‐and‐pencil” K‐SADS (Kaufman et al., 1997) and includes the essential components of the “paper‐and‐pencil” K‐SADS: an unstructured introductory interview, a diagnostic screening interview, supplements to finalize the criteria required for each diagnosis (Townsend et al., 2020). K‐SADS‐COMP questionnaires are completed without a clinician's supervision on‐site or remotely. Diagnoses are provided for both parent‐report and child‐report results.

A list of K‐SADS diagnoses we included under the umbrella terms of “depression”, “ADHD” and “anxiety” are available in Supporting Information S6.

**HBN: K‐SADS‐based clinician diagnosis.** We used the clinician consensus diagnosis, based on clinician‐administered K‐SADS‐COMP. The K‐SADS‐COMP consists of parent and child interviews, which are used to derive automated diagnoses; administration in HBN is performed by a licensed clinician. A team of clinicians then reviews the accumulated materials and agrees on a DSM‐5 diagnosis. HBN provides the KSADS‐COMP interview data, the automatically generated diagnoses, and the clinicians' consensus diagnoses (Alexander et al., 2017).

**BHRC: DAWBA‐based clinician diagnosis.** BHRC also provides a clinicians' consensus diagnosis. It is based on another automated structured interview ‐ the Development and Well‐being Assessment (DAWBA) (Goodman et al., 2000). DAWBA includes structured answers as well as verbatim responses of any reported problems. All questions are closely related to DSM‐IV diagnostic criteria. The study uses the Brazilian Portuguese version of DAWBA (Fleitlich, Bilyk and Goodman, 2004). It was administered to biological parents of the participating children, and no interviews were conducted with the children. DAWBA structured interviews are administered by lay interviewers, and the rating procedures are performed by nine certified child psychiatrists. Cases in which there was a disagreement about diagnoses were discussed between two child psychiatrists until consensus was achieved (Salum et al., 2015).

### Study design

#### Sample size and sample size rationale

Samples sizes vary between datasets and analyses as shown in Table 1. In each case we used all applicable positive cases and included enough controls to achieve a sample with 10% positive cases (based on rates in the literature (e.g. (Ebesutani et al., 2017; Li et al., 2021). If there were not enough controls to create a sample with 10% positive cases, we used all controls that were available. See Supporting Information S1 for detailed sample size rationale and power calculations.

**TABLE 1:** Demographics of ABCD, HBN, BHRC samples.

	Dataset	Reporter	N (female)	Age (std)	Dep	No dep	Anx	ADHD
1: Sensitivity	ABCD	Parent	300 (156)	9.54 (0.50)	30	270	40	30
	ABCD	Child	1189 (576)	9.46 (0.51)	119	1070	23	0
	HBN	Clinician	214 (89)	10.45 (1.44)	76	138	46	55
	BHRC	Clinician	710 (327)	10.43 (1.28)	71	639	86	87
2a: Specificity	ABCD	Parent	300 (125)	9.48 (0.51)	30	270	162	169
	ABCD	Child	197 (81)	9.50 (0.51)	119	78	92	0
	HBN	Clinician	760 (247)	10.31 (1.41)	76	784	345	628
	BHRC	Clinician	465 (219)	10.42 (1.28)	71	394	232	234
2b: Strict specificity	ABCD	Parent	NA	NA	NA	NA	NA	NA
	ABCD	Child	183 (75)	9.50 (0.51)	105	78	78	0
	HBN	Clinician	NA	NA	NA	NA	NA	NA
	BHRC	Clinician	390 (186)	10.37 (1.30)	39	351	186	197

Abbreviations: ABCD, adolescent brain cognitive development; ADHD, attention deficit/hyperactivity disorder; Anx, anxiety; BHRC, Brazilian high risk cohort study; Dep, depression; HBN, healthy brain network.

Due to insufficient data, it was not possible to test Strict Specificity in the HBN and on the parent‐report ABCD data. We only tested it in child‐report ABCD and BHRC.

### Statistical methods

We evaluated the performance of the CBCL‐Aff with the Area Under the Receiver Operator Curve (AUCROC). AUCROC is a performance metric for classification problems that accounts for the trade‐off between sensitivity and specificity. The Receiver Operator Characteristic (ROC) is a plot of the true positive rate (TPR; sensitivity; recall) against the false positive rate (FPR; 1 ‐ specificity) at a range of classification thresholds, and the AUCROC is the area beneath it. The AUCROC ranges from 0 to 1, with 0.5 being chance performance (i.e. a useless model), AUCROC greater than 0.8 is commonly defined as “excellent” performance (e.g. (Ferdinand, 2008)). An AUCROC of 1.0 corresponds to a perfect classifier.

We assessed the significance of comparisons between the observed AUCROCs and the threshold AUCROCs with a one‐sided bootstrap *t*‐test (as preregistered) and a two‐sided bootstrap *t*‐test (as presented in the main paper). We calculated the AUCROC for each of 1000 bootstrap iterations and then obtained the one‐sided *p*‐values by calculating the number of AUCROCs that were above the predefined threshold of “goodness”, and dividing that number by the number of bootstraps +1 for the original arrangement of the data (1001 in this case).

For AUROC, we used the threshold value of 0.8 to reject the H0. This corresponds to a “excellent” performance, as used in several studies (e.g. (Ferdinand, 2008)).

We also looked at optimal diagnostic thresholds as exploratory analysis (Table S7 in Supporting Information S7).

### Deviations from pre‐registrations

The parent‐report ABCD analysis and the HBN/BRHC analysis were preregistered separately. The child‐report ABCD analysis, the Brief Problem Monitor analysis, and the analyses of anxious/depressed and withdrawn/depressed scales were not preregistered and should be treated as exploratory.

The optimal diagnostic CBCL‐Aff threshold values and the corresponding confusion matrices found in Supporting Information S6 were exploratory and were not preregistered.

While we proposed one‐sided bootstrap *t*‐tests in our preregistrations, the CBCL‐Aff frequently outperformed our expectations and we were able to confirm via bootstrap *t*‐test that it was better than the threshold we specified. We present two‐sided *p*‐values in the results section for the convenience of the reader. Our original, one‐sided *p*‐values for the test that the CBCL‐Aff's performance was below threshold are available, Table S8 in Supporting Information S8.

While our preregistration specifies the age for ABCD from 9 to 11, during the analysis of our data, we noticed a few participants in the ABCD baseline wave that were aged 8. In order to maximize our sample size, we included these participants, so the age range of participants in the ABCD analyses is 8–11.

While our registrations for HBN and BHRC specify ages 9–13, we meant greater than 9 and less than 13, which we now realize should have been described as “aged 9–12 years old”. We have kept the analysis restricted to 8–12 years old in these datasets as this more closely matches the age range in the baseline ABCD sample.

### Code availability

All code for our analyses and figures is available online at https://github.com/transatlantic‐comppsych/CBCL_Aff_Validation.

## RESULTS

Our sample was balanced gender‐wise and included participants with the mean age 9.46–10.45, depending on the dataset and the analysis (Table 1).

We tested Sensitivity (depressed vs. not depressed adolescents), Specificity (depressed with or without comorbidity vs. non‐depression diagnoses), and Strict Specificity (depressed and without comorbidity vs. non‐depression diagnoses) on three datasets: ABCD, HBN and BHRC.

### ABCD results

**FIGURE 2:**
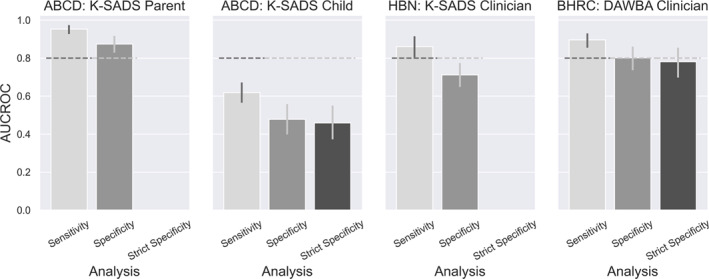
AUCROC results for all datasets (ABCD, HBN, BHRC) and all analyses (Sensitivity, Specificity, Strict Specificity). ABCD, Adolescent Brain Cognitive Development; AUCROC, Area under the Receiver Operator Curve; BHRC, Brazilian High Risk Cohort study; HBN, Healthy Brain Network; DAWBA, the Development and Well‐being Assessment, K‐SADS, the Kiddie Schedule for Affective Disorders and Schizophrenia.

**FIGURE 3:**
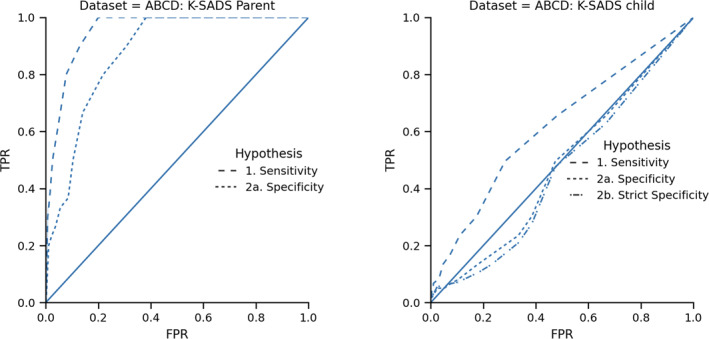
AUCROC plots for ABCD analyses, comparing CBCL‐Aff against parent‐report K‐SADS (left panel) and child‐report K‐SADS (right panel). ABCD, Adolescent Brain Cognitive Development; FPR, False Positive Rate; K‐SADS, the Kiddie Schedule for Affective Disorders and Schizophrenia; TPR, True Positive Rate.

In our initial preregistered analysis, we examined the performance of the CBCL‐Aff for predicting diagnosis based on the parent‐report K‐SADS (Table 2, Figure 2, left panel of Figure 3). For both analyses, CBCL‐Aff performed significantly better than our pre‐defined threshold (Sensitivity: AUCROC = 0.95 (95% CI: 0.93, 0.97), two‐sided *p* < 0.001; Specificity: AUCROC = 0.87 (95% CI: 0.83, 0.91), two‐sided *p* < 0.001) (Table 2).

**TABLE 2:** AUCROC and *p*‐values obtained from all datasets on CBCL‐Aff measure.

	Dataset	Reporter	N Pos	N Neg	Thresh.	AUCROC	*p*‐value
1: Sensitivity	ABCD	Parent	30	270	0.8	0.953 (0.927, 0.974)	<0.001*
	ABCD	Child	119	1071	0.8	0.619 (0.565, 0.672)	<0.001*
	HBN	Clinician	76	138	0.8	0.861 (0.804, 0.915)	0.036*
	BHRC	Clinician	71	639	0.8	0.896 (0.855, 0.930)	<0.001*
2a: Specificity	ABCD	Parent	30	270	0.8	0.874 (0.828, 0.917)	<0.001*
	ABCD	Child	119	78	0.8	0.478 (0.398, 0.558)	<0.001*
	HBN	Clinician	76	684	0.8	0.712 (0.648, 0.774)	0.006*
	BHRC	Clinician	71	394	0.8	0.802 (0.738, 0.860)	0.931
2b: Strict specificity	ABCD	Parent	NA	NA	NA	NA	NA
	ABCD	Child	105	78	0.8	0.459 (0.372, 0.550)	<0.001*
	HBN	Clinician	NA	NA	NA	NA	NA
	BHRC	Clinician	39	351	0.8	0.781 (0.697, 0.855)	0.653

*Note*: Reported *p*‐values are two‐sided. AUCROC = Area Under the Receiver Operator Curve. AUCROC values are reported with 95% confidence intervals, in parentheses. Indicated *p*‐values are obtained by bootstrapping. *p* < 0.001 indicates that none of the bootstrapped values crossed the pre‐set threshold. Significant *p*‐values (<0.05) are indicated with an asterisk (*). The definitions of the positive and negative groups are laid out in Figure 1.

Following the strong performance of the CBCL‐Aff for predicting parent‐report K‐SADS, we compared the CBCL‐Aff to the child‐report K‐SADS in an exploratory analysis (Table 2, Figure 2 right panel of Figure 3). Here the CBCL‐Aff was far below our threshold (Sensitivity: AUCROC = 0.62 (95% CI: 0.57, 0.67), two‐sided *p* < 0.001; Specificity: AUCROC = 0.48 (95% CI: 0.40, 0.56), two‐sided *p* < 0.001, Strict Specificity AUCROC = 0.46 (95% CI: 0.37, 0.55), two‐sided *p* < 0.001).

CBCL‐AD and CBCL‐WD performed worse than CBCL‐Aff (Tables S10‐1 and S10‐2 in Supporting Information S10). On parent‐report K‐SADS, both CBCL‐AD and CBCL‐WD performed significantly better than threshold on Sensitivity. At the same time, their Specificity results were not statistically different from threshold. On child‐report K‐SADS, CBCL‐AD and CBCL‐WD performed significantly worse than threshold on Sensitivity, Specificity, and Strict Specificity (Tables S10‐1 and S10‐2 in Supporting Information S10).

While the three scales share few items (Supporting Information S5), they are all strongly correlated in ABCD's baseline wave (CBCL‐Aff with CBCL‐AD *r* = 0.768 *p* < 0.001; CBCL‐Aff with CBCL‐WD *r* = 0.696, *p* < 0.001; CBCL‐WD with CBCL‐AD *r* = 0.664, *p* < 0.001).

To sum up, in **ABCD**, the concordance between the CBCL‐Aff and parent‐report KSADS was better than the concordance between the CBCL‐Aff child‐report KSADS, on all tests. We turned to the HBN and BHRC datasets to reconcile these results since they have consensus clinicians' diagnosis.

### HBN results

In **HBN**, CBCL‐Aff was a good predictor for clinicians' diagnosis, AUCROC for Sensitivity = 0.86 (95% CI: 0.80, 0.92), two‐sided *p*‐value = 0.036. However, CBCL‐Aff was not a good differentiator between depression and other forms of psychopathology (Specificity AUCROC = 0.71 (95% CI: 0.65, 0.77), two‐sided *p*‐value = 0.006; AUCROC was significantly lower than threshold; also see Table 2, Figure S11‐1 in Supporting Information S11).

### BHRC results

In **BHRC**, CBCL‐Aff performed well on all three analyses. For Sensitivity, AUCROC values were significantly above the threshold (AUCROC = 0.90 (95% CI: 0.86, 0.93), two‐sided *p*‐value <0.001). For Specificity and Strict Specificity, AUCROC value was not significantly different from the pre‐defined threshold (Specificity: AUCROC = 0.80 (95% CI: 0.74, 0.86), two‐sided *p*‐value 0.931; Strict Specificity: AUCROC = 0.78 (95% CI: 0.70, 0.86), two‐sided *p*‐value = 0.653; also see Table 2, Figure S11‐2 in Supporting Information S11).

The CBCL‐Aff generally performs well as a predictor of parent and clinician depression diagnosis in the ABCD, HBN, and BHRC datasets, but is notably poor as a predictor of depression based on child self‐report of symptoms in ABCD. K‐SADS depression diagnosis is notably discordant between children and parents in ABCD, with children and parents agreeing that the child has depression in only 4 cases in the baseline wave (Table 3). Given this discordance, it would be informative to compare a self‐report measure such as the Youth Self‐Report (Achenbach & Rescorla, 2001), but the only self‐report measure available thus far in ABCD is the Brief Problem Monitor (BPM). The BPM's (Achenbach et al., 2011) internal problem scale had similar sensitivity for the child‐report K‐SADS depression diagnosis as the CBCL‐Aff did for the parent‐report K‐SADS diagnosis child‐report K‐SADS Sensitivity AUCROC = 0.91 (95% CI: 0.88, 0.94), but was much less specific Specificity AUCROC = 0.52 (95% CI: 0.43, 0.60), Strict Specificity AUCROC = 0.44 (95% CI: 0.35, 0.53) (See, Figure S12 in Supporting Information S12, Table S12 in Supporting Information S12).

**TABLE 3:** Agreement rates between depression diagnoses in ABCD based on K‐SADS.

	Parent report +	Parent report −
Child report +	4	70
Child report −	25	6060

## DISCUSSION

Psychometrically sound measures enable meaningful analyses (Flake & Fried, 2020). This work evaluated the validity of CBCL‐Aff as a measure of depression in the popular ABCD dataset. We compared the CBCL‐Aff measurement against the parent‐report and child‐report K‐SADS. The analysis on the child‐report K‐SADS was not preregistered and therefore should be treated as exploratory. We found that in ABCD, CBCL‐Aff was a good predictor of parent‐report K‐SADS, but performed below chance in predicting child‐report K‐SADS. This pattern of results could be explained purely by within rater correspondence. This idea is supported by our results on child‐report BPM, which had similar sensitivity for the child‐report KSADS as parent‐report CBCL‐Aff had for parent‐report KSADS. The lower specificity for BPM as compared to CBCL‐Aff is unsurprising given the small number of relevant items in the BPM. To resolve the rater correspondence problem, we validated the CBCL‐Aff against diagnoses from clinically trained external observers in the HBN and BHRC datasets.

In HBN, which used clinician consensus diagnosis based on a mix of parent‐ and child‐report data, CBCL‐Aff performed well on Sensitivity (depressed vs. not depressed) but performed worse on Specificity (depressed vs. other psychopathology). In BHRC, which used clinician diagnosis based on a parent‐report measure (Development and Well‐being Assessment (DAWBA) (Goodman et al., 2000)), CBCL‐Aff performed well on Sensitivity, but its performance was not significantly different from the “goodness” thresholds on Specificity and Strict Specificity. Overall, if we based our assessment on clinician diagnosis, then these results suggest that CBCL‐Aff is a valid continuous measure of depression severity in the ABCD data.

This conclusion disagrees with our hypothesis, that parent‐report CBCL‐Aff would be a poor predictor of the K‐SADS. On the other hand, our results agree with the previous research, which had successfully validated CBCL on other datasets and populations similar to ours in age (Ebesutani et al., 2010; Kazdin et al., 1983; Lacalle et al., 2012; Leung et al., 2006; Nakamura et al., 2009; Skarphedinsson et al., 2021).

In general, these studies agreed on high rates of parent‐child disagreement (Bilenberg, 1999; Kazdin et al., 1983), which we also observed. The direction of disagreement, however, varied. Kazdin et al. (1983) concluded that children may underestimate their symptomatology, while Bilenberg (1999) observed that children reported more emotional problems than their parents and teachers. Leung et al. (2006) concluded that CBCL was better at screening externalizing problems, which are commonly known to be reported by parents more often than children. Bilenberg (1999) saw children and parents agreeing less on the externalizing behavior (and more on emotional problems).

The problem of informant discrepancy (De Los Reyes et al., 2011) is widely recognized by depression researchers (Abate et al., 2018; Angold et al., 1987; Baumgartner et al., 2020; De Los Reyes & Kazdin, 2005; Kashani et al., 1985), see (De Los Reyes et al., 2015) for a review. Importantly, it impacts judgements on treatment outcome (Goolsby et al., 2018). However, the reason parents and children disagree generally remain unclear (De Los Reyes & Kazdin, 2005). Some suggest it reflects parent's incomplete insights into their children's internal states (Kim et al., 2016), or children's failure to report their symptoms (Kazdin et al., 1983; Orvaschel et al., 1981), which leads problems in children to be missed (Makol & Polo, 2018).

Children can effectively monitor their feelings (Carroll & Steward, 1984) and recognize emotions (Vasa et al., 2006). At the same time, they may struggle to understand belief‐based emotions as compared to false beliefs, even if they were asked to identify their own emotions (Bender et al., 2011) (although the sample in this study was younger than ours). This might influence children's ability to identify problems in their affect and behavior. This could support the idea of giving parents of younger children more weight when deciding on the child's diagnosis. On the other hand, some research (Abate et al., 2018) warns against using parent‐report measurements as a proxy for children's affective state. Finally, another view is to avoid considering one perspective (parent's or child's) as more accurate. In this view, parents and children are endorsing different aspects of the child's dysfunction (Kazdin et al., 1983) and therefore should both be taken into account (Kazdin et al., 1983).

Moreover, previous research suggests that agreement between parents and children on the child's depression symptomatology may vary as a function of age (Berg‐Nielsen et al., 2003; Kazdin et al., 1983). Our sample consisted of children aged 8–11 (ABCD), or 8–12 (HBN, BHRC). While having a psychiatric disorder in childhood (up to 12 years old) or adolescence (13–18 years old) is a potent risk factor for being diagnosed in the adult years (Copeland et al., 2013), biological factors underlying depression vary across the lifespan (Kaufman et al., 2001). Depressed children and adolescents differ from depressed adults on measures of basal cortisol secretion, corticotropin stimulation post‐corticotropin releasing hormone (CRH) infusion, response to several serotonergic probes, immunity indices, and efficacy of tricyclic medications (Kaufman et al., 2001).

Since CBCL‐Aff reflects parent assessment, identifying neural correlates of the CBCL‐Aff in children is relying on external assessment of largely subjective, internal states. This could introduce several problems. First, the difference in whether the parents report more or less internalizing problems may be rooted in the affective problems the parent themselves is experiencing (Berg‐Nielsen et al., 2003), confounding parental affective state with the measure of their children's symptoms. Moreover, differences between adult depression and child/adolescent depression mean that what parents report as symptoms of depression might be impacted by the specific features of adult, as opposed to child, depression. Parents might lack insight in subjective experience of depression due to the differences in biological processes underlying adult and child depression.

Second, there is an important implication of our results on the use of CBCL‐Aff as a measure of depression in imaging studies. If researchers use CBCL‐Aff in studies that look for neural correlates of depression, they might find correlates of scores that in many cases contradict the report given by the individual.

Third, researchers should be cautious to use “depression” as a term judiciously and consider the age range they are investigating as this may have implications about what measures/features predict it best.

Finally, ABCD is an unselected cohort aiming to approach population representativeness, whereas the other two cohorts oversampled psychopathology. A population sample is bound to have few cases and therefore be more imbalanced than one where cases were oversampled for psychopathology. This imbalance could affect the estimates of classification accuracy. We account for this by balancing our data to have 10% ratio of positive cases in all datasets, if the amount of available data allows for it. Also, population samples generally suffer from attrition/undersampling of cases with severity being an indicator of non‐selection/attrition. Hence, inferences drawn from a population sample would be restricted to less severe cases that are not representative of what one might encounter in a clinical situation. Hence, complementing a population sample with a sample that oversampled psychopathology can be seen as beneficial. Finally, it is conceivable that in a population sample, the classification accuracy of cases is larger because of the differences between people with depression and controls—controls will score around zero. By contrast, in enriched samples, classification accuracy might be expected to be smaller because cases there will be people who may not meet threshold for depression, but still score relatively highly on depression (e.g. someone with severe anxiety may not meet full criteria for depression but have a high score).

### Limitations

Our study had the following limitations. First, we did not perform item‐theoretic analyses to determine if CBCL‐Aff is consistently sensitive across a range of severity, this may be an important question to answer in future work. Second, the DAWBA measure which was used to inform clinician‐report diagnosis in the BHRC dataset was only administered to parents. The other dataset with clinician‐report measures (HBN), however, used K‐SADS which was administered to both parents and children. Third, the Youth‐Self Report data are not available in the baseline wave, so as a self‐report measure, we used the Brief Problem Monitor, which is significantly different from CBCL‐Aff in the questions that it covers. Fourth, we compared CBCL‐Aff against K‐SADS, which is an imperfect measure itself. There is no commonly accepted gold standard in the field. Our findings suggest that CBCL‐Aff is valid in the ABCD sample only as long as K‐SADS diagnoses are valid. Given the concerns about validity of K‐SADS, validating CBCL scales against K‐SADS diagnoses is suboptimal. Finally, there were some participants with missing K‐SADS/DAWBA diagnoses, which we excluded from our sample.

## CONCLUSION

Based on clinician diagnosis, the parent‐report CBCL‐Aff is a valid continuous measure of depression in 8–12 year olds, though it does disagree with self‐report of symptoms.

## AUTHOR CONTRIBUTIONS

**Marie Zelenina**: Formal analysis; visualization; writing ‐ original draft; writing ‐ review and editing. **Daniel S. Pine**: Conceptualization; funding acquisition; methodology; supervision; writing ‐ review and editing. **Argyris Stringaris**: Conceptualization; funding acquisition; methodology; supervision; writing ‐ review and editing. **Dylan M. Nielson**: Conceptualization; formal analysis; methodology; supervision; visualization; writing ‐ original draft; writing ‐ review and editing.

## CONFLICT OF INTEREST STATEMENT

The authors declare no conflict of interest.

## ETHICAL CONSIDERATIONS

All approvals have been obtained by investigators of corresponding datasets. Please See Supporting Information S3 for details.

## Supporting information

Supporting Information S1

## Data Availability

The data that support the findings of this study are available from the NIMH Data Archive (ABCD dataset), Child Mind Institute (HBN dataset) and the authors of the BHRC dataset. Restrictions apply to the availability of these data, which were used under license for this study. Data are available: ABCD dataset ‐ https://nda.nih.gov/abcd/; HBN dataset ‐ https://data.healthybrainnetwork.org/main.php; BHRC dataset ‐ from the authors https://osf.io/ktz5h/wiki/home/ with the permission of the authors.
