# Supplementary material for: Validation of CBCL depression scores of adolescents in three independent datasets
Source: JCPP Adv. 2025 Jan 29;5(3):e12298. doi: 10.1002/jcv2.12298 (PMC12446721; doi:10.1002/jcv2.12298)
Supplement: Supplementary file 1 — Supporting Information S1 [file JCV2-5-e12298-s001.docx]

Table of Contents

[Supporting Information S1: Power calculations (simulations) and sample sizes 3](#_Toc161054962)

[Sample size 3](#_Toc161054963)

[Parent-report ABCD 3](#_Toc161054964)

[Child-report ABCD 6](#_Toc161054965)

[HBN 9](#_Toc161054966)

[BHRC 13](#_Toc161054967)

[Power calculations 17](#_Toc161054968)

[Supporting Information S2: Enrolment details and exclusion and 19](#_Toc161054969)

[inclusion criteria for ABCD, HBN, BHRC 19](#_Toc161054970)

[ABCD 19](#_Toc161054971)

[HBN 19](#_Toc161054972)

[BHRC 20](#_Toc161054973)

[Supporting Information S3: Ethics 22](#_Toc161054974)

[Supporting Information S4: Missing data 23](#_Toc161054975)

[Supporting Information S5: CBCL items included in the CBCL-Aff, anxious/depressed, and withdrawn/depressed scales 24](#_Toc161054976)

[Supporting Information S6: Diagnoses comprising ”depression”, ”ADHD” and ”anxiety” 26](#_Toc161054977)

[Supporting Information S7: Optimal CBCL diagnostic thresholds and confusion matrices for ABCD data 28](#_Toc161054978)

[Supporting Information S8: Results with one-sided p-values 30](#_Toc161054979)

[Supporting Information S9: Comparison of analysis of boys and girls separately. 31](#_Toc161054980)

[Supporting Information S10: CBCL-WD and CBCL-AD 33](#_Toc161054981)

[Supporting Information S11: HBN and BHRC results 35](#_Toc161054982)

[Supporting Information S12: Brief Problem Monitor 37](#_Toc161054983)

# Supporting Information S1: Power calculations (simulations) and sample sizes

## Sample size

### Parent-report ABCD

For **parent-report ABCD**, we had 11747 participants in the baseline data. After removing participants with missing data, we had n = 6186.

For the Sensitivity and the Specificity, we had the number of children meeting the diagnostic criteria n = 30 (0.3% of the baseline data). We then limited the number of children in our negative sample to reach 10% positive ratio, which was based on literature (e.g. Ebesutani et al., 2017, Li et al., 2021). Our final samples for Sensitivity and Specificity was n = 300 (Figures S1, S2). We did not Strict Specificity on parent-report ABCD.


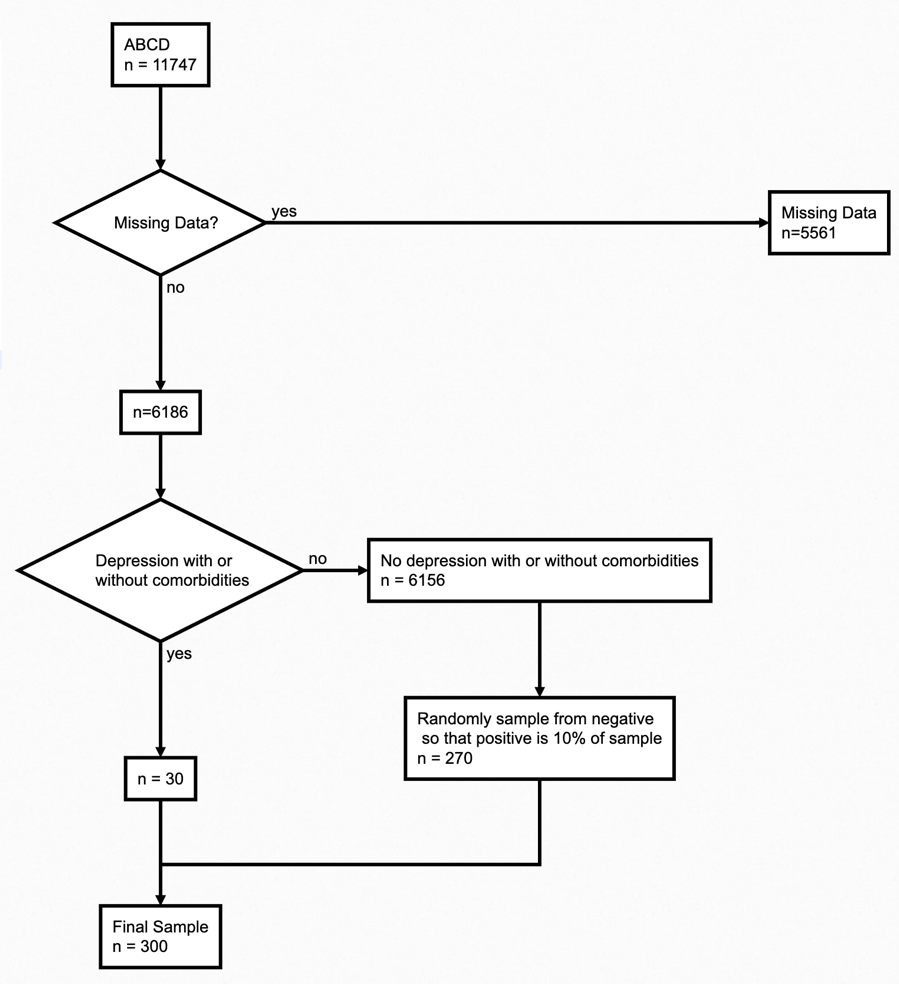


Figure S1. Flow chart of sample selection for Sensitivity on parent-report ABCD data.


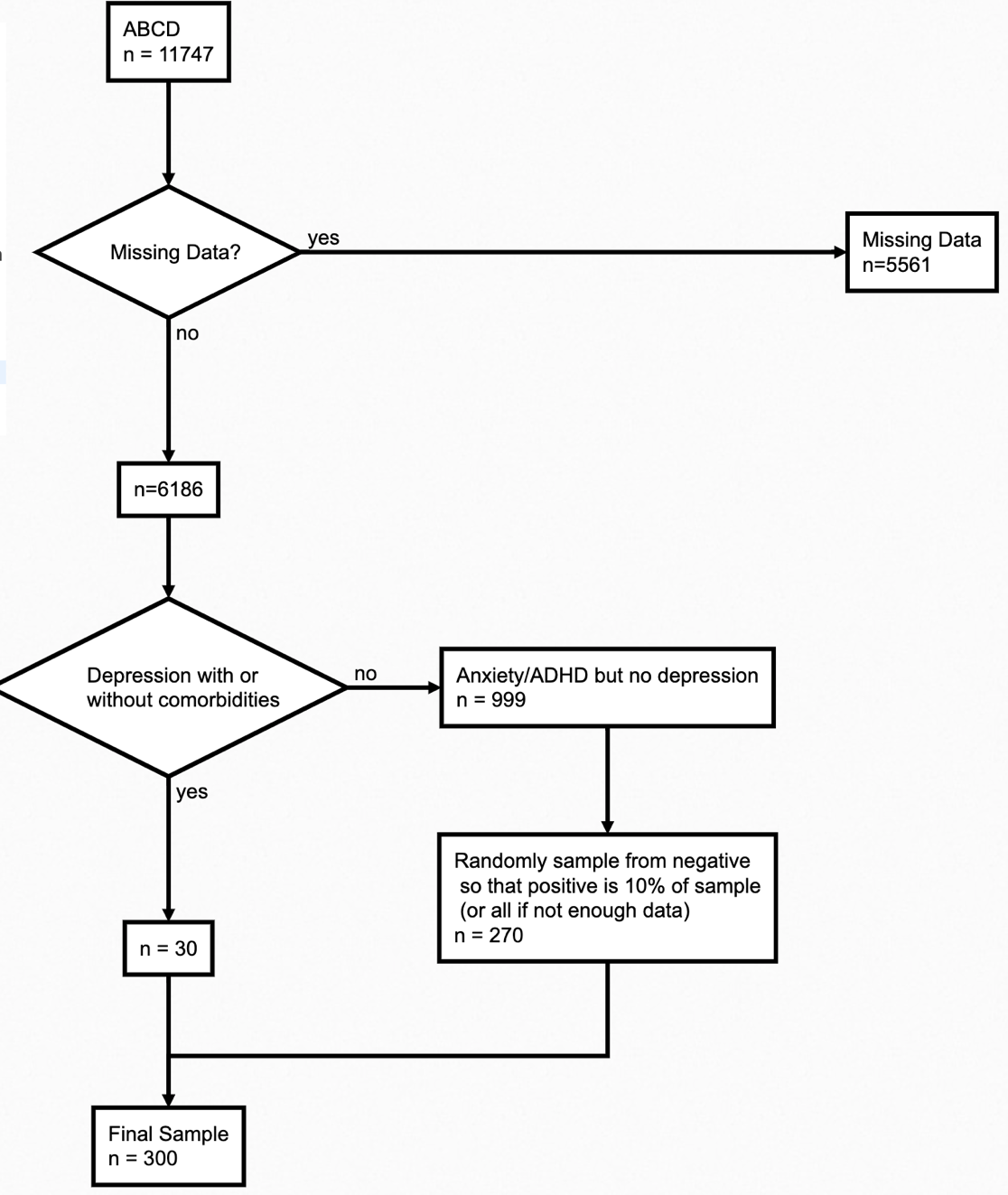


Figure S2. Flow chart of sample selection for Specificity on parent-report ABCD data.

### Child-report ABCD

For **child-report ABCD**, we followed a similar procedure.

We had 11812 participants in the baseline data, and after removing 20 lines of missing data, we had the baseline sample of n = 11792.

For the Sensitivity analysis, we had 119 children meeting the diagnostic criteria for depression (1% of baseline data). We then limited the number of children in our negative sample to reach 10% positive case ratio. Our final sample for Sensitivity consisted of n = 1189 children (Figure S3).

For the Specificity analysis, we did not have enough children without depression but with another form of psychopathology (anxiety, ADHD) to form the 90% of the sample. As such, we used all available negative data (n=78), and had the positive-negative ratio as 60%-40%, with the final sample consisting of n = 197 children (Figure S4).

For the Strict Specificity analysis, we had 105 children meeting the diagnostic criteria for depression, but not meeting diagnostic criteria for ADHD or anxiety (0.9% of baseline data), which formed our positive sample. We did not have enough children in our negative sample (children with anxiety and/or ADHD but without depression) to reach 10%-90% positive-negative ratio, and as such, we used all available children in our negative sample (n = 78) and reached a 57%-43% positive-negative ratio. Our final sample consisted of n = 183 children (Figure S5).


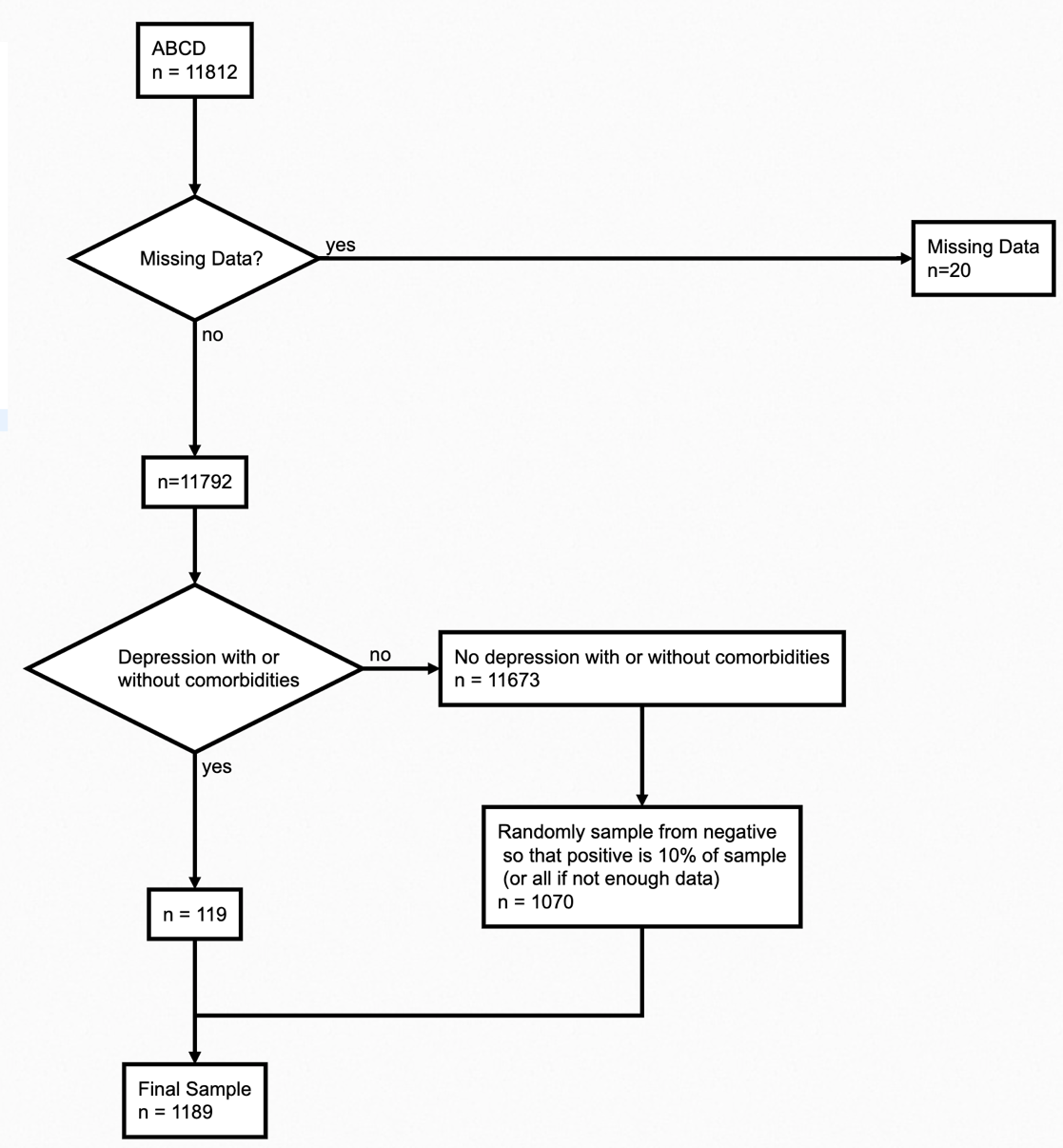


Figure S3. Flow chart of sample selection for Sensitivity on child-report ABCD data.


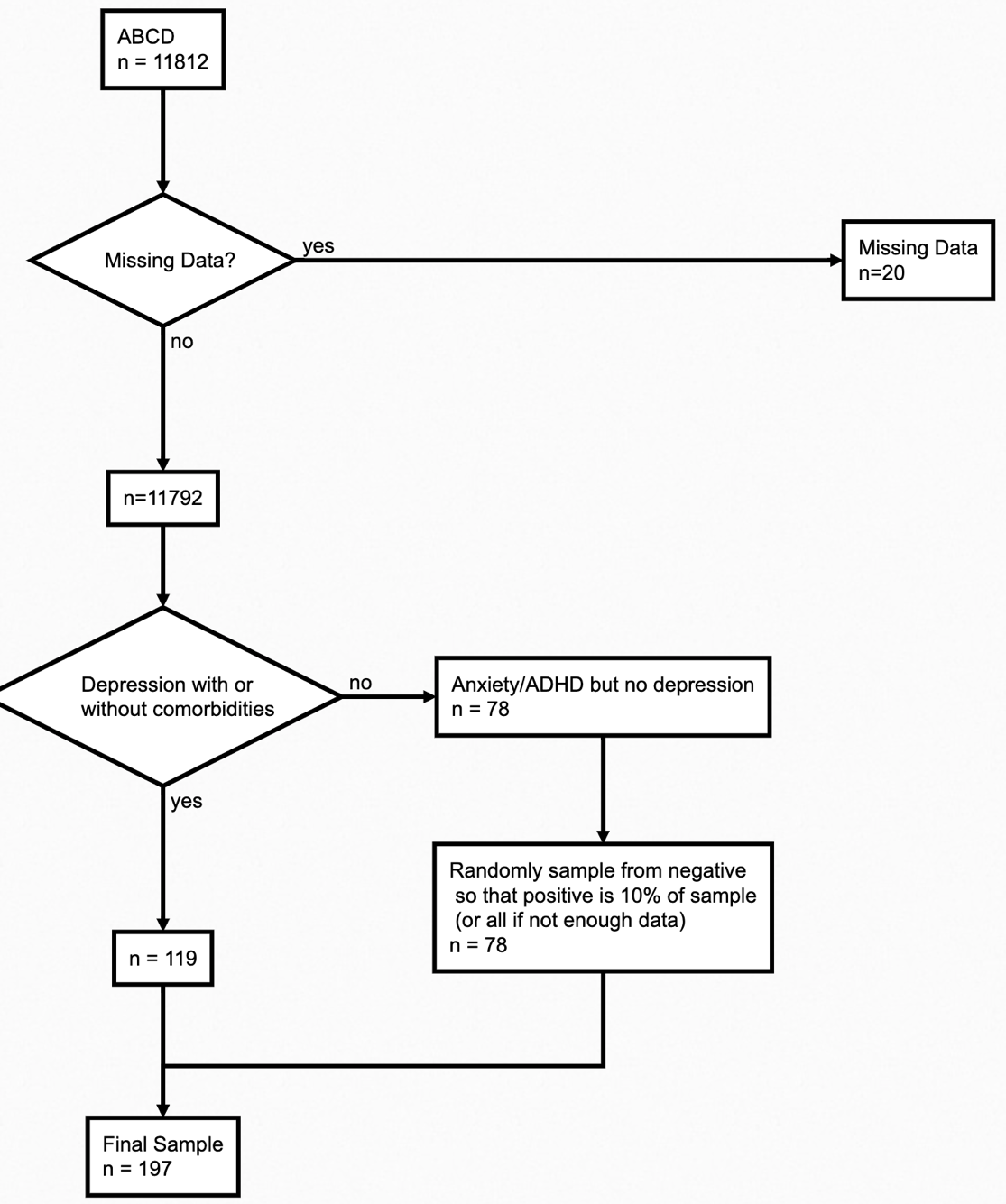
Figure S4. Flow chart of sample selection for Specificity on child-report ABCD data.


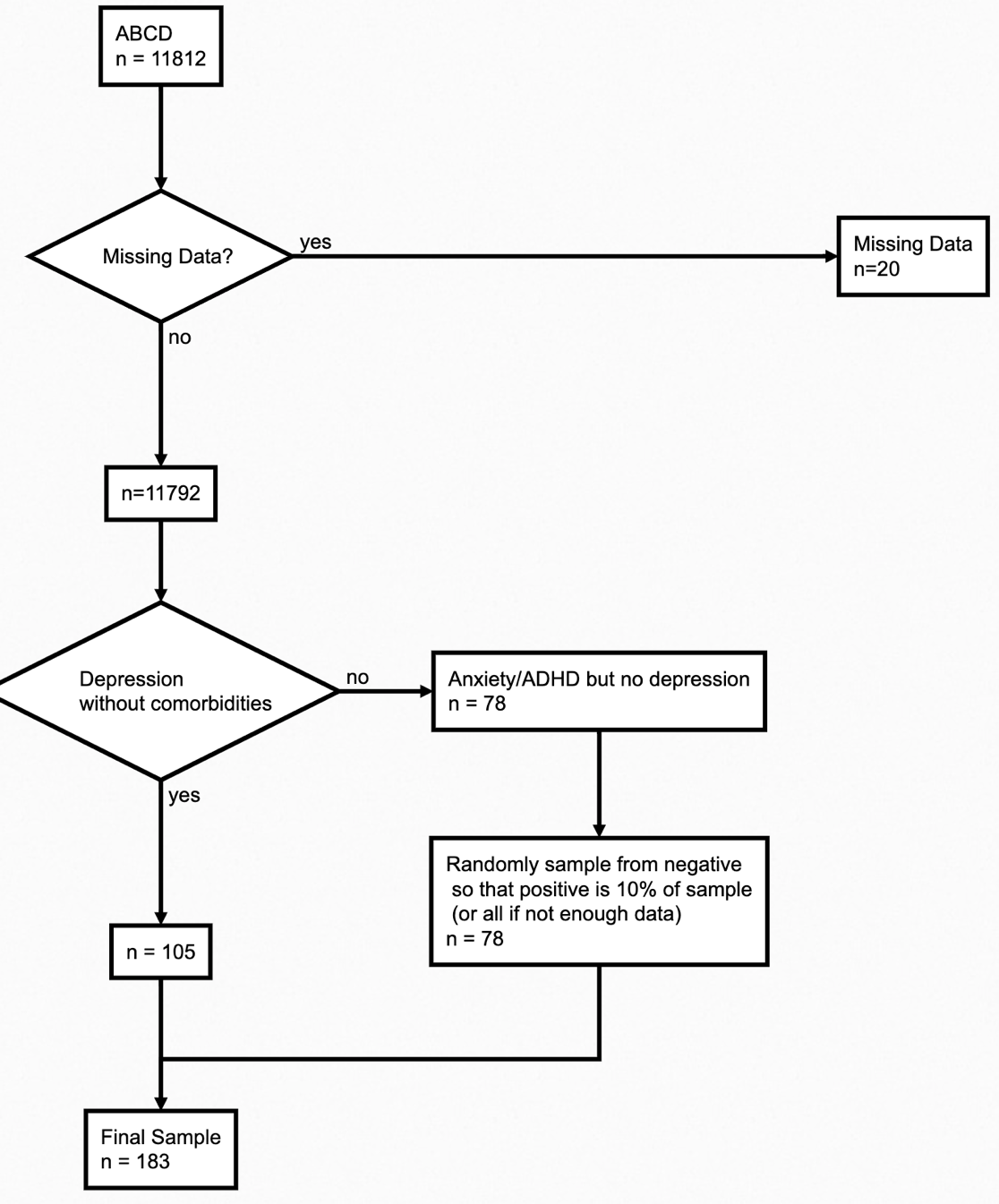


Figure S5. Flow chart of sample selection for Strict Specificity on child-report ABCD data.

###

### HBN

For **HBN**, we followed a similar procedure.

We had 6182 participants in the data we downloaded. After removing participants with missing data (n = 2562), we had a sample of n = 3620.

For the Sensitivity and Specificity analyses, we had n = 76 participants with a positive diagnosis of depression (1.2% of baseline data).

For the Sensitivity analysis, we did not have enough participants to reach 10%-90% positive-negative split, so we used all participants in the negative sample (n = 138), reaching the overall sample of n = 214 and a 35%-65% positive-negative split (Figure S6).

For the Specificity analysis, we had enough participants to reach a 10% positive ratio. The overall sample size was n = 760 (Figure S7).

We did not test Strict Specificity on HBN data.


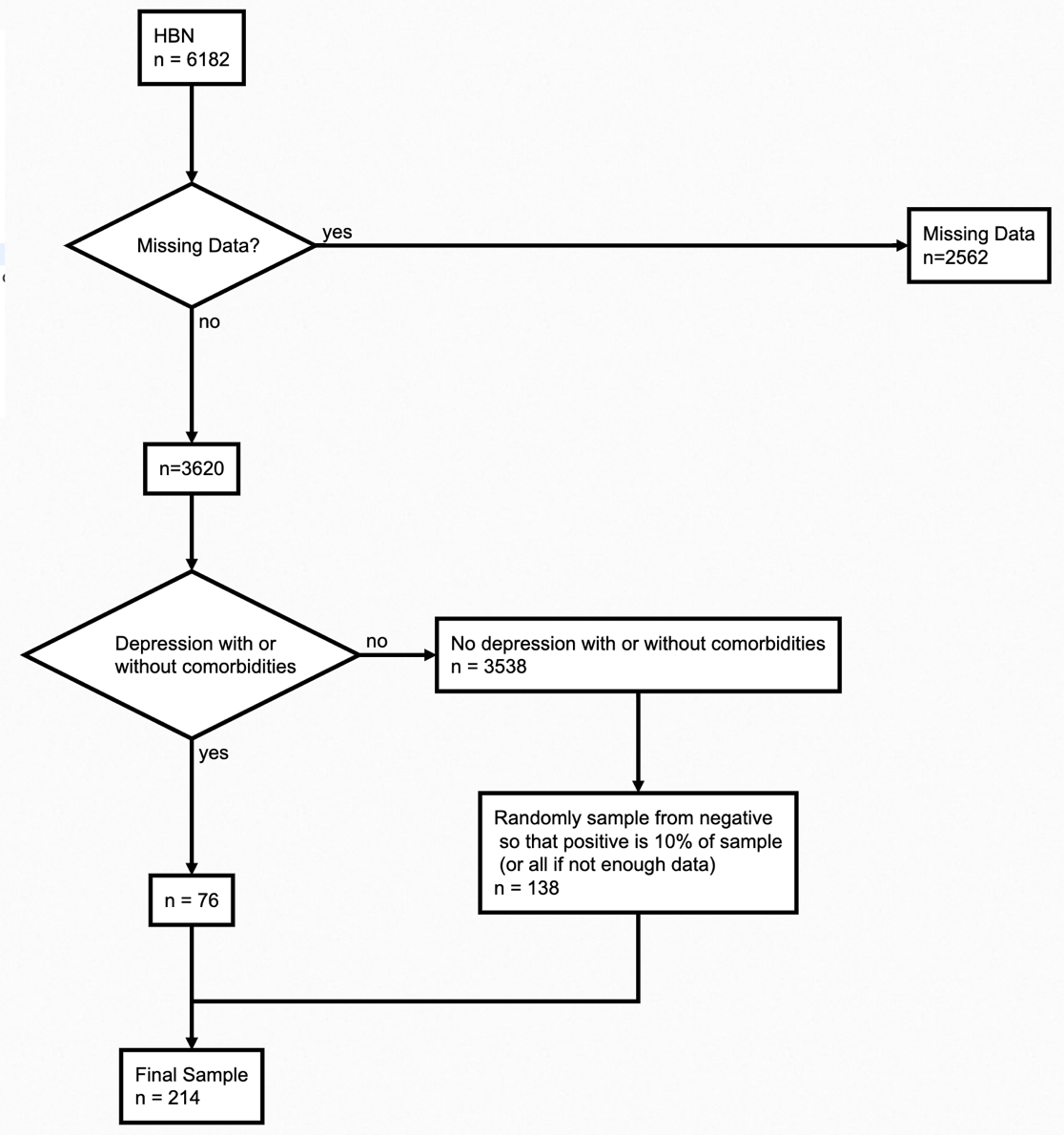


Figure S6. Flow chart of sample selection for Sensitivity analysis on HBN data.


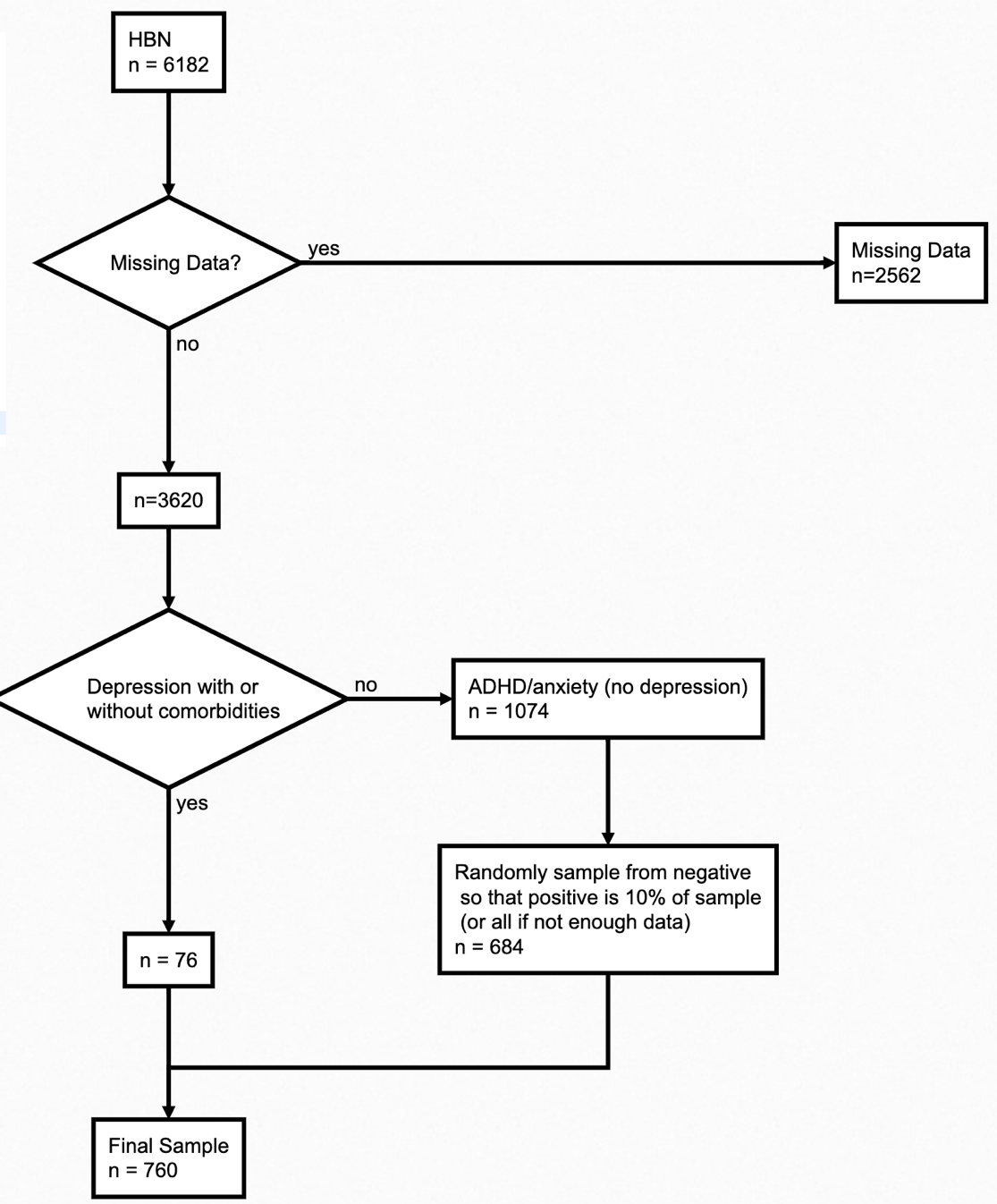


Figure S7. Flow chart of sample selection for Specificity analysis on HBN data.

### BHRC

For **BHRC data**, we followed the same procedure, with an additional step of limiting the participants by age (8-13 years old) (Figure S9-S11), because the BHRC sample has mixed age of participants.

We had n = 7533 participants in the data we downloaded. After removing participants with missing data (n = 27), we had n = 7506 participants.

We then selected participants that were within the age range we were interested in (n = 2636).

For the Sensitivity analysis, we had 71 participants in the positive sample (0.9% of baseline data). To reach the 10%-90% positive-negative ratio, we limited the number of participants in the negative sample to n = 639, resulting in the overall sample of n = 710 (Figure S8).

For the Specificity analysis, we also had 71 participants in the positive sample, but not enough participants in the negative sample to reach the 10%-90% ratio. We used all available participants in the negative sample (n = 394), resulting in the overall sample of n = 465 and 15%-85% positive-negative ratio (Figure S9).

For the Strict Specificity analysis, we had n = 39 participants with a diagnosis of depression but without a diagnosis of anxiety and/or ADHD, and we limited the number of participants without depression but with anxiety and/or ADHD to reach the 10-90% positive-negative ratio. Our final sample had 351 participants in the negative sample and 390 participants overall (Figure S10).


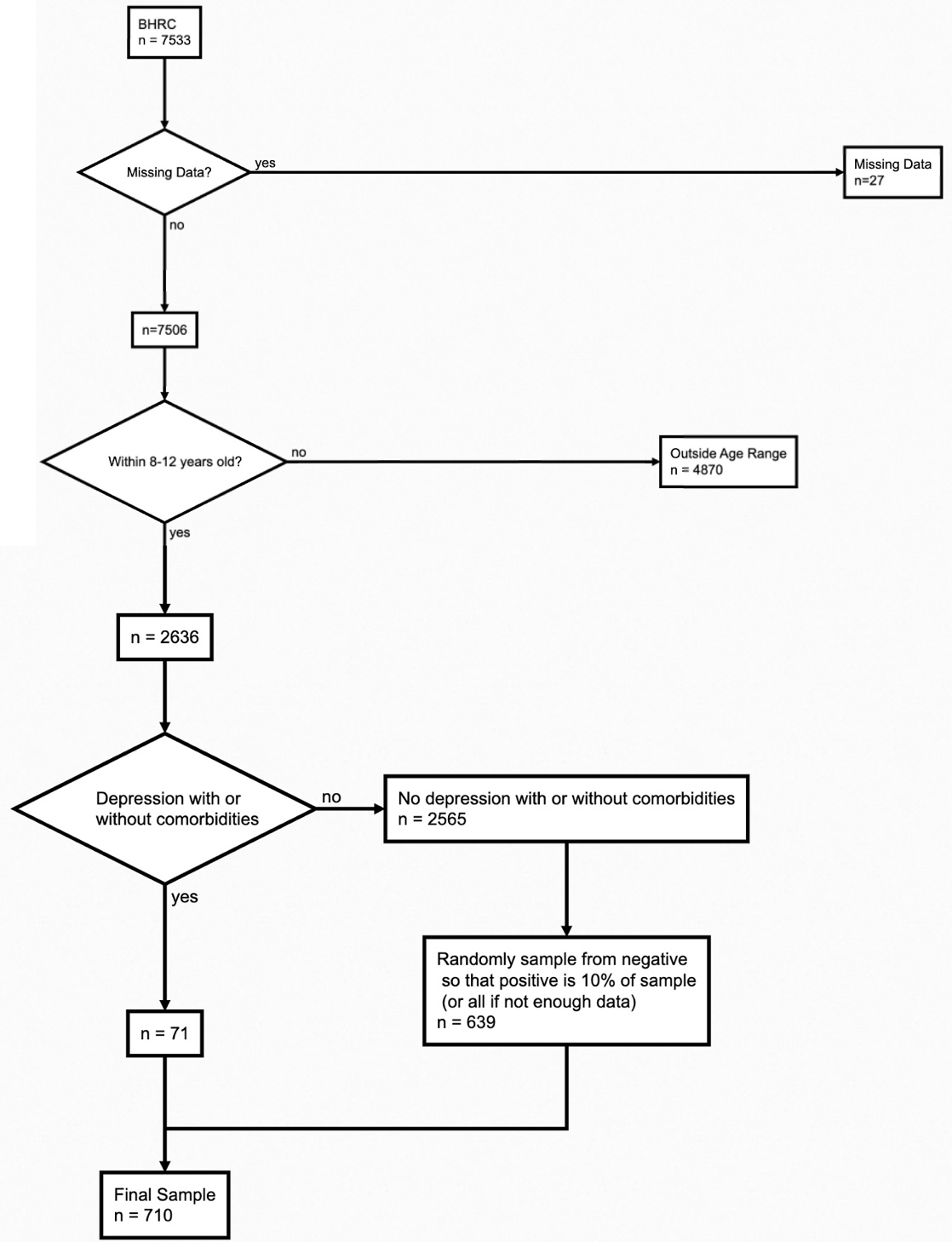


Figure S8. Flow chart of sample selection for Sensitivity analysis on BHRC data.


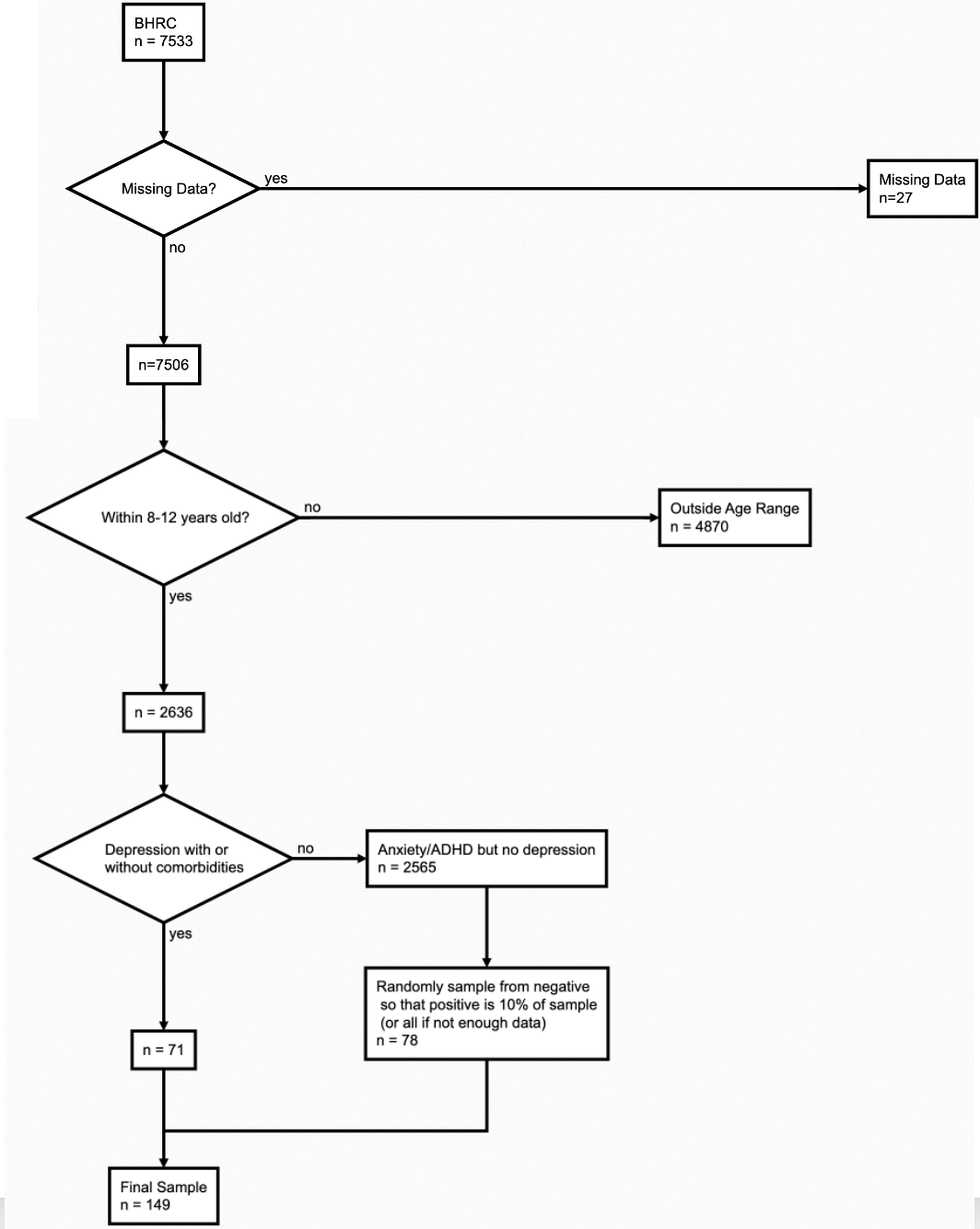


Figure S9. Flow chart of sample selection for Specificity analysis on BHRC data.


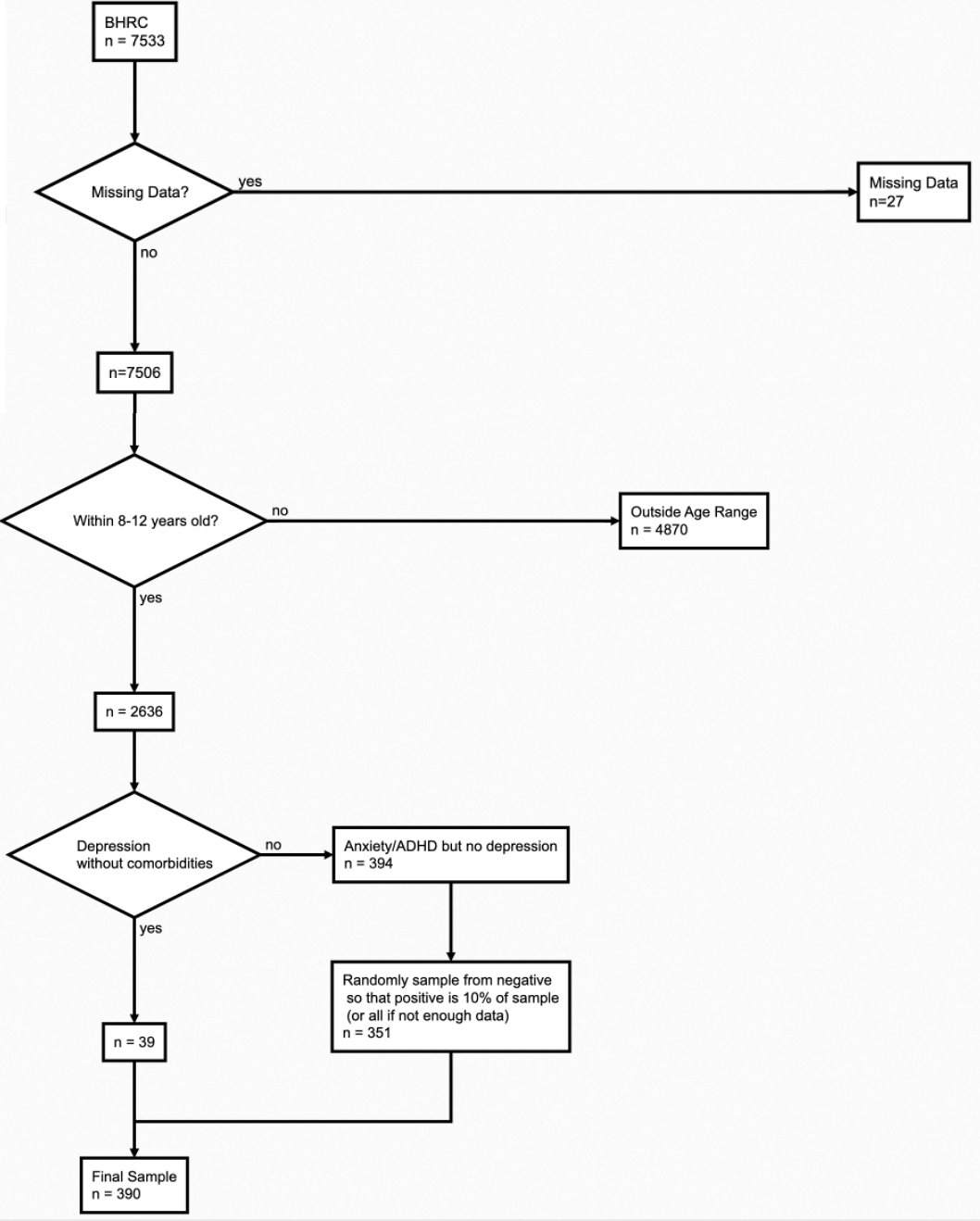


Figure S10. Flow chart of sample selection for Strict Specificity analysis on BHRC data.

## Power calculations

As power is a function of effect size, which in turn depends on the population AUC, we ran 500 simulations to determine the range of the population AUCs at which we have an 80% power to correctly reject the null hypothesis. In each of the 500 simulations, we first simulated a large (n = 100,000) population, matched to our true (ABCD/HBN/BHRC) data in the positive case rate. We linked two marginal distributions (Gaussian, representing the continuous CBCL-Aff score, and Bernoulli, representing the binary K-SADS diagnosis) with a Gaussian copula with correlation drawn randomly from a uniform distribution between 0 and 1. Next, we calculated the population AUC and drew a random sample (n = overall sample size) from the population. On that subsample, we performed a one-sided bootstrap t-test testing the hypothesis of the AUC being less than 0.8 within the sample. We calculated the cumulative power as a function of population AUC and determined the population AUC at which we had 80% power to correctly reject the null hypothesis.

From this estimation, we found the value of population AUC which allows us an 80% power to correctly reject the null. The threshold AUC values are presented in Table S1.

As most our estimations resulted in the threshold AUCROC = 0.8, and to ensure comparability of our results, we then set AUCROC thresholds for all datasets and all hypotheses to 0.8. We report the actual power estimation numbers for AUCROC = 0.8 in Table S1.

|  | **Dataset** | **Reporter** | **N** | **N Pos** | **Pos rate** | **AUCROC**  **thresh.** | **Power at AUCROC = 0.8** |
| --- | --- | --- | --- | --- | --- | --- | --- |
| **1: SSensitivity** | **ABCD** | **Parent** | 300 | 30 | 10% | 0.77 | 74% |
|  | **ABCD** | **Child** | 1189 | 119 | 10% | 0.82 | 87% |
|  | **HBN** | **Clinician** | 214 | 76 | 35% | 0.80 | 80% |
|  | **BHRC** | **Clinician** | 710 | 71 | 10% | 0.81 | 84% |
| **2a: Specificity** | **ABCD** | **Parent** | 300 | 30 | 10% | 0.77 | 74% |
|  | **ABCD** | **Child** | 197 | 119 | 60% | 0.8 | 80% |
|  | **HBN** | **Clinician** | 760 | 76 | 10% | 0.8 | 80% |
|  | **BHRC** | **Clinician** | 465 | 71 | 15% | 0.81 | 82% |
| **2b: Strict Specificity** | **ABCD** | **Parent** | NA | NA | NA | NA | NA |
|  | **ABCD** | **Child** | 183 | 105 | 57% | 0.83 | 86% |
|  | **HBN** | **Clinician** | NA | NA | NA | NA | NA |
|  | **BHRC** | **Clinician** | 390 | 39 | 10% | 0.78 | 74% |

**Table S1 Power estimations for ABCD, HBN, and BHRC datasets. Pos = positive, thresh. = threhold.**

# Supporting Information S2: Enrolment details and exclusion and inclusion criteria for ABCD, HBN, BHRC

## ABCD

Exclusion criteria for the children were as follows: child not fluent in English, parent not fluent in English or Spanish, child has major medical or neurological conditions, gestational age < 28 weeks or birthweight < 1200g, contraindications to MRI scanning, a history of traumatic brain injury, a current diagnosis of schizophrenia, moderate/severe autism spectrum disorder, intellectual disability, or alcohol/substance use disorder [Michelini et al., 2019].

The sociodemographic factors that informed the recruitment included age, gender, race and ethnicity, socioeconomic status, and urbanicity [Garavan et al., 2018].

**Informed consent**

Most ABCD research sites rely on a central Institutional Review Board (cIRB) at the University of California, San Diego for the ethical review and approval of the research protocol, with a few sites obtaining local IRB approval. Parental consent and child assent is obtained at each visit.

## HBN

HBN used a community-referred recruitment model, with the goal of capturing the natural heterogeneity and impairment in the sample. The project was advertised thorough the community, to educators, local care providers and parents. Participants were offered a monetary compensation. Participants (or their legal guardians) go through a pre-screening phone interview. This interview aims to identify safety concerns. As a general rule, psychiatric, medical, or neurological illnesses do not exclude participation. A full list of inclusion and exclusion criteria is as follows:

**HBN inclusion criteria:**

Male or female ages 5–21 years; Adults must have capacity to understand the study and provide informed consent; Children ages 5–17 must have the capacity to provide assent (must speak in simple, but full (3+ word) sentences at the Kindergarten level) and parent/guardian must have the capacity to sign informed consent; Participants must be fluent in English. Children who are fluent in English but have parents who speak Spanish can be enrolled upon availability of Spanish-speaking personnel. **HBN exclusion criteria:**

Serious neurological (specific or focal) disorders preventing full participation in the protocol; Acute encephalopathy (brain dysfunction) caused by an injury to the brain or disease; Known neurodegenerative disorder (e.g., Huntington’s Disease, ALS, MS, Cerebral Palsy); Hearing or visual impairment that prevents participation in study-related tasks (child can participate if vision or hearing is corrected with devices); ecent diagnosis (within the past 6 months) of Schizophrenia, Schizoaffective Disorder, or Bipolar Disorder; Manic or psychotic episode within the past 6 months without current, ongoing treatment; New onset (within the last 3 months) of suicidality or homicidality for which there is no current, ongoing treatment; History of lifetime substance dependence requiring chemical replacement therapy; Acute intoxication at time of any study visit.

**Informed consent**

The study was approved by the Chesapeake Institutional Review Board (https://www.chesapeakeirb.com/). Prior to conducting the research, written informed consent is obtained from participants ages 18 or older. For participants younger than 18, written consent is obtained from their legal guardians and written assent obtained from the participant.

## BHRC

BHRC dataset aimed to include children at high risk of developing a psychiatric disorder and thus screening was performed to select a sub-sample of children with “high risk” for developing mental disorders. High risk labelling included family history and early psychiatric symptoms. Data was collected from public schools close to the research centers with more than 1000 students in the age of interest. Participants were recruited at schools. Participants were not paid for participation, although compensation of transport expenses for offered for some participants. Eligibility criteria were as follows: (1) the child was registered by a biological parent that was a primary carer and could provide sufficient information about the children’s behavior; and (2) the child is 6–12 years old at enrollment.

**Informed consent**

Written consent was obtained from parents of the participants and from participants who were able to read, write and understand the written consent. From others, verbal agreement was obtained.

# Supporting Information S3: Ethics

Most ABCD research sites rely on a central Institutional Review Board (cIRB) at the University of California, San Diego for the ethical review and approval of the research protocol, with a few sites obtaining local IRB approval. Parental consent and child assent is obtained at each visit.

The HBN study was approved by the Chesapeake Institutional Review Board (https://www.chesapeakeirb.com/). Prior to conducting the research, written informed consent is obtained from participants ages 18 or older. For participants younger than 18, written consent is obtained from their legal guardians and written assent obtained from the participant.

BHRC was approved by the ethics committee of the University of São Paulo (IORG0004884/National Council of Health Registry number (CONEP): 15.457/Project IRB registration number: 1132/08). Written consent was obtained from parents of the participants and from participants who were able to read, write and understand the written consent. From others, verbal agreement was obtained.

# Supporting Information S4: Missing data

In ABCD with child-report KSADS, data were missing for 6 out of 10 anxiety diagnoses: Other Specified Anxiety Disorder (Panic Disorder, impairment, does not meet full criteria), F41.8; Panic Disorder (F41.0) PRESENT; Other Specified Anxiety Disorder (Agoraphobia, impairment, does not meet full criteria), F41.8; Separation Anxiety Disorder (F93.00) PRESENT; Other Specified Anxiety Disorder (Separation Anxiety Disorder, impairment, does not meet full criteria), F41.8; Specific Phobia PRESENT (F40.2XX), and for all ADHD diagnoses. We excluded the missing diagnoses from consideration. In HBN data we assumed ”no diagnosis” was meant whenever diagnosis from a single clinician was missing for a subject.

# Supporting Information S5: CBCL items included in the CBCL-Aff, anxious/depressed, and withdrawn/depressed scales

|  | cbcl-aff | anx/dep | with/dep |
| --- | --- | --- | --- |
| “There is very little they enjoy” | + | - | - |
| “Cries a lot” | + | + | - |
| “Deliberately harms self or attempts suicide” | + | - | - |
| “Doesn't eat well” | + | - | - |
| “Feels worthless or inferior” | + | + | - |
| “Feels too guilty” | + | + | - |
| “Overtired without good reason” | + | - | - |
| “Sleeps less than most kids “ | + | - | - |
| “Sleeps more than most kids” | + | - | - |
| “Talks/thinks suicide” | + | - | - |
| “Trouble sleeping” | + | - | - |
| “Underactive, slow moving, or lacks energy” | + | - | - |
| “Unhappy, sad, or depressed” | + | + | + |
| “Complains of loneliness” | - | + | - |
| “Impulsive or acts without thinking” | - | + | - |
| “Feels they need to be perfect” | - | + | - |
| “Feels or complains that no one loves them” | - | + | - |
| “Feels others are out to get them” | - | + | - |
| “Nervous, highstrung, or tense” | - | + | - |
| “Too fearful or anxious” | - | + | - |
| “Self-conscious or easily embarrassed” | - | + | - |
| “Suspicious” | - | + | - |
| “Worries” | - | + | - |
| “Would rather be alone than with others” | - | - | + |
| “Refuses to talk” | - | - | + |
| “Secretive, keeps things to self” | - | - | + |
| “Too shy or timid” | - | - | + |
| “Underactive, slow moving, or lacks energy” | - | - | + |
| “Withdrawn, doesn't get involved with others” | - | - | + |
| “Stares blankly” | - | - | + |
| “Sulks a lot” | - | - | + |

Table S5. CBCL items included in the CBCL-Aff, anxious/depressed, and withdrawn/depressed scales

# Supporting Information S6: Diagnoses comprising ”depression”, ”ADHD” and ”anxiety”

We defined ”depression”, ”ADHD” and ”anxiety” as matching the following the following KSADS diagnoses:

- Depression
  1. Major Depressive Disorder Present
  2. Major Depressive Disorder, Current, in Partial Remission
  3. Persistent Depressive Disorder (Dysthymia), Present
  4. Persistent Depressive Disorder (Dysthymia), In Partial Remission
  5. Unspecified Depressive Disorder, Current
- ADHD
  1. Attention-Deficit/Hyperactivity Disorder, Present
  2. Attention-Deficit/Hyperactivity Disorder, in Partial Remission
  3. Unspecified Attention-Deficit/Hyperactivity Disorder
- Anxiety
  1. Generalized Anxiety Disorder, Present
  2. Specific Phobia, Present
  3. Panic Disorder, Present
  4. Separation Anxiety Disorder, Present
  5. Social Anxiety Disorder, Present
  6. Other Specified Anxiety Disorder (Panic Disorder, impairment, does not meet full criteria)
  7. Other Specified Anxiety Disorder (Agoraphobia, impairment, does not meet full criteria)
  8. Other Specified Anxiety Disorder (Separation Anxiety Disorder, impairment, does not meet full criteria)
  9. Other Specified Anxiety Disorder (Social Anxiety Disorder, impairment, does not meet minimum duration)
  10. Other Specified Anxiety Disorder (Generalized Anxiety Disorder, impairment, does not meet minimum duration)

# Supporting Information S7: Optimal CBCL diagnostic thresholds and confusion matrices for ABCD data

CBCL-Aff provides a continuous score of depression, which is then transferred into a binary diagnosis based on a diagnostic threshold X (if the score is above X, the child is diagnosed as having depression). For exploratory purposes, we defined the optimal diagnostic threshold by maximizing the Euclidian distance from the AUCROC plot to the optimal AUCROC value (top left corner of the plotting space). At the optimal diagnostic threshold value, we report the confusion matrix: true positives, true negatives, false positives, and false negatives.

Optimal CBCL diagnostic thresholds and confusion matrices for ABCD data are summarized in Table S7.

|  | **Dataset** | **Diagn. thresh.** | **TP** | **TN** | **FP** | **FN** |
| --- | --- | --- | --- | --- | --- | --- |
| **1: Sensitivity** | **ABCD-parent** | 4 | 27 | 235 | 35 | 3 |
|  | **ABCD-child** | 2 | 59 | 768 | 303 | 60 |
| **2a: Specificity** | **ABCD-parent** | 5 | 24 | 211 | 59 | 6 |
|  | **ABCD-child** | 2 | 59 | 41 | 37 | 60 |
| **2b: S Specificity** | **ABCD-parent** | NA | NA | NA | NA | NA |
|  | **ABCD-child** | 2 | 50 | 41 | 37 | 55 |

Table S7: Optimal CBCL diagnostic thresholds that maximize the AUCROC and confusion matrix values for parent-report and child-report ABCD. TP = True Positive, TN = True Negative, FP = False Positive, FN = False Negative

# Supporting Information S8: Results with one-sided p-values

|  | **Dataset** | **Reporter** | **N Pos** | **N Neg** | **AUCROC** | **p-value** |
| --- | --- | --- | --- | --- | --- | --- |
| **1: Sensitivity** | **ABCD** | **Parent** | 30 | 270 | 0.953 (0.927, 0.974) | 1.0 |
|  | **ABCD** | **Child** | 119 | 1071 | 0.619 (0.565, 0.672) | <0.001* |
|  | **HBN** | **Clinician** | 76 | 138 | 0.861 (0.804, 0.915) | 0.982 |
|  | **BHRC** | **Clinician** | 71 | 639 | 0.896 (0.855, 0.930) | 1.0 |
| **2a: Specificity** | **ABCD** | **Parent** | 30 | 270 | 0.874 (0.828, 0.917) | 1.0 |
|  | **ABCD** | **Child** | 119 | 78 | 0.478 (0.398, 0.558) | <0.001* |
|  | **HBN** | **Clinician** | 76 | 684 | 0.712 (0.648, 0.774) | 0.003* |
|  | **BHRC** | **Clinician** | 71 | 394 | 0.802 (0.738, 0.860) | 0.534 |
| **2b: Strict Specificity** | **ABCD** | **Parent** | NA | NA | NA | NA |
|  | **ABCD** | **Child** | 105 | 78 | 0.459 (0.372, 0.550) | <0.001* |
|  | **HBN** | **Clinician** | NA | NA | NA | NA |
|  | **BHRC** | **Clinician** | 39 | 351 | 0.781 (0.697, 0.855) | 0.327 |

Table S8: AUCROC and p-values obtained from all datasets (ABCD, HBN, BHRC). Reported p-values re one-sided. AUCROC = Area Under the Receiver Operator Curve. Indicated p-values are obtained by bootstrapping. P = 1 indicates that all the bootstrapped values were above the pre-set threshold, p = 0 indicates that none of the bootstrapped values were above the pre-set threshold. Significant p-values (*<*0.05) are indicated with an asterisk (*).

# Supporting Information S9: Comparison of analysis of boys and girls separately.

| **Analysis** | **Reporter** | **N Pos** | **N Neg** | **AUCROC** | **p-value** |
| --- | --- | --- | --- | --- | --- |
| **1: Sensitivity** | **Parent** | 19 | 137 | 0.951 (0.909, 0.986) | <0.001* |
|  | **Child** | 43 | 387 | 0.627 (0.561, 0.686) | <0.001* |
| **2a: Specificity** | **Parent** | 19 | 106 | 0.849 (0.760, 0.927) | 0.246 |
|  | **Child** | 43 | 38 | 0.503 (0.395, 0.615) | <0.001* |
| **2b: Strict Specificity** | **Parent** | NA | NA | NA | NA |
|  | **Child** | 37 | 38 | 0.491 (0.381, 0.599) | <0.001* |

Table S9-1: AUCROC and p-values obtained from ABCD dataset, girls only. Reported p-values are two-sided. AUCROC = Area Under the Receiver Operator Curve. AUCROC values are reported with 95% confidence intervals, in parentheses. Indicated p-values are obtained by bootstrapping. Significant p-values (*<*0.05) are indicated with an asterisk (*).

| **Analysis** | **Reporter** | **N Pos** | **N Neg** | **AUCROC** | **p-value** |
| --- | --- | --- | --- | --- | --- |
| **1: Sensitivity** | **Parent** | 11 | 99 | 0.951 (0.904, 0.984) | <0.001* |
|  | **Child** | 76 | 537 | 0.626 (0.561, 0.688) | <0.001* |
| **2a: Specificity** | **Parent** | 11 | 99 | 0.848 (0.763, 0.926) | 0.266 |
|  | **Child** | 76 | 40 | 0.506 (0.395, 0.615) | <0.001* |
| **2b: Strict Specificity** | **Parent** | NA | NA | NA | NA |
|  | **Child** | 68 | 40 | 0.492 (0.381, 0.603) | <0.001* |

Table S9-2: AUCROC and p-values obtained from ABCD dataset, boys only. Reported p-values are two-sided. AUCROC = Area Under the Receiver Operator Curve. AUCROC values are reported with 95% confidence intervals, in parentheses. Indicated p-values are obtained by bootstrapping. Significant p-values (*<*0.05) are indicated with an asterisk (*).

# Supporting Information S10: CBCL-WD and CBCL-AD

| **Analysis** | **Reporter** | **N Pos** | **N Neg** | **AUCROC** | **p-value** |
| --- | --- | --- | --- | --- | --- |
| **1: Sensitivity** | **Parent** | 30 | 270 | 0.911 (0.844, 0.958) | 0.010* |
|  | **Child** | 119 | 1071 | 0.628 (0.577, 0.682) | <0.001* |
| **2a: Specificity** | **Parent** | 30 | 270 | 0.808 (0.727, 0.878) | 0.334 |
|  | **Child** | 119 | 78 | 0.438 (0.355, 0.531) | <0.001* |
| **2b: Strict Specificity** | **Parent** | NA | NA | NA | NA |
|  | **Child** | 105 | 78 | 0.416 (0.334, 0.494) | <0.001 |

Table S10-1: AUCROC and p-values obtained from ABCD dataset on CBCL Anxious/Depressed measure. Reported p-values are two-sided. AUCROC = Area Under the Receiver Operator Curve. AUCROC values are reported with 95% confidence intervals, in parentheses. Indicated p-values are obtained by bootstrapping. Significant p-values (*<*0.05) are indicated with an asterisk (*).

| **Analysis** | **Reporter** | **N Pos** | **N Neg** | **AUCROC** | **p-value** |
| --- | --- | --- | --- | --- | --- |
| **1: Sensitivity** | **Parent** | 30 | 270 | 0.914 (0.868, 0.955) | <0.001* |
|  | **Child** | 119 | 1071 | 0.632 (0.582, 0.681) | <0.001* |
| **2a: Specificity** | **Parent** | 30 | 270 | 0.825 (0.749, 0.887) | 0.130 |
|  | **Child** | 119 | 78 | 0.469 (0.384, 0.550) | <0.001* |
| **2b: Strict Specificity** | **Parent** | NA | NA | NA | NA |
|  | **Child** | 105 | 78 | 0.451 (0.365, 0.533) | <0.001* |

Table S10-2: AUCROC and p-values obtained from ABCD dataset on CBCL Withdrawn/Depressed measure. Reported p-values are two-sided. AUCROC = Area Under the Receiver Operator Curve. AUCROC values are reported with 95% confidence intervals, in parentheses. Indicated p-values are obtained by bootstrapping. Significant p-values (*<*0.05) are indicated with an asterisk (*).

# Supporting Information S11: HBN and BHRC results

**
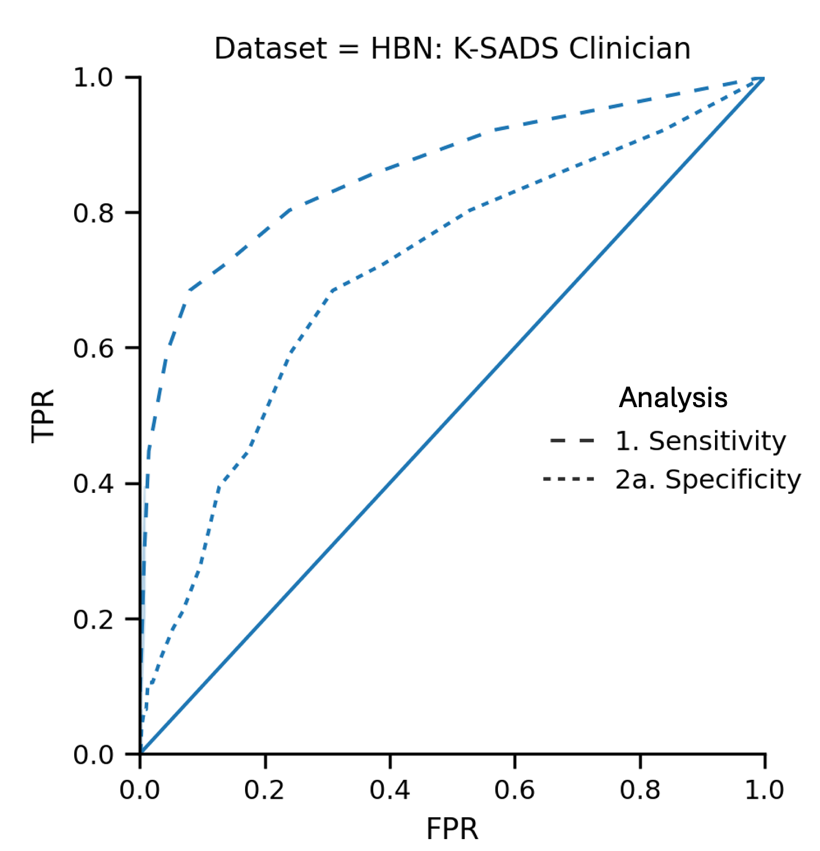
**

Figure S11-1: Receiver-Operator Curves (ROC) for the HBN data. Analysis 1: Sensitivity; adolescents with vs. without a current diagnosis of depression. Analysis 2a: Specificity; adolescents with depression vs. adolescents without depression but with another form of psychopathology. Analysis 2b: Strict Specificity; adolescents with depression but without any other form of psychopathology vs. adolescents without depression but with another form of psychopathology.


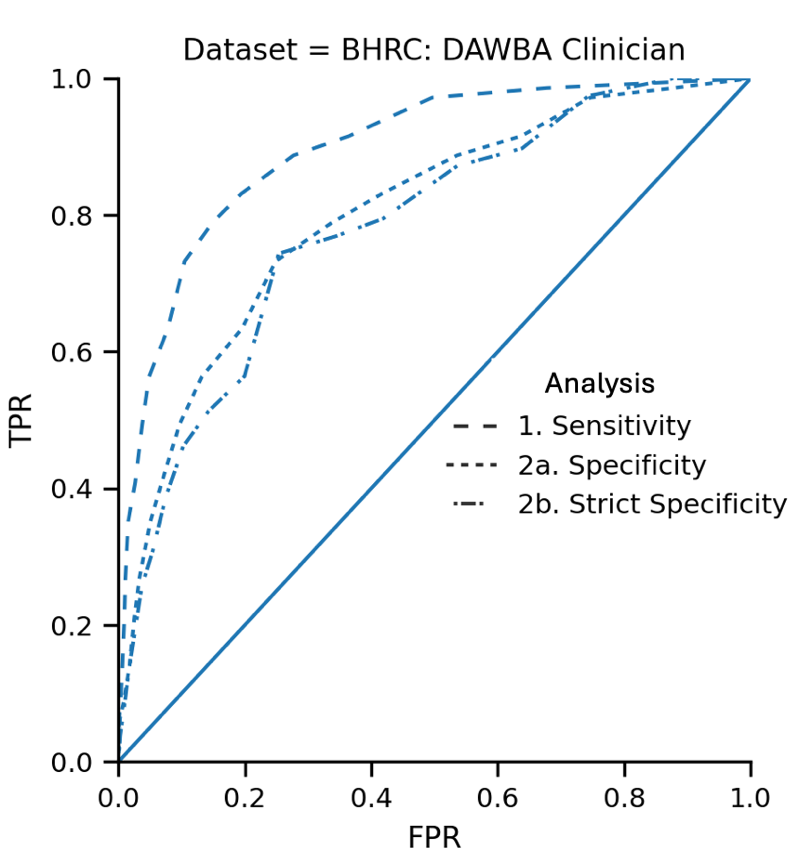


Figure S11-2: Receiver-Operator Curves (ROC) for the BHRC data. Analysis 1: Sensitivity; adolescents with vs. without a current diagnosis of depression. Analysis 2a: Specificity; adolescents with depression vs. adolescents without depression but with another form of psychopathology. Analysis 2b: Strict Specificity; adolescents with depression but without any other form of psychopathology vs. adolescents without depression but with another form of psychopathology.

# Supporting Information S12: Brief Problem Monitor

**
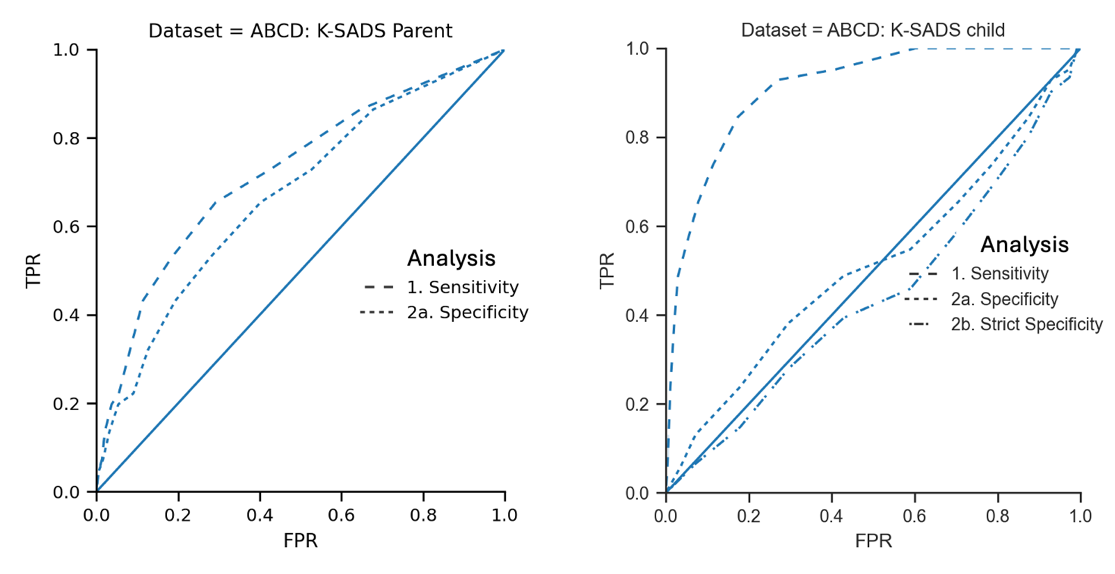
**

Figure S12. AUCROC plots for Brief Problem Monitor.

| **Analysis** | **Reporter** | **N Pos** | **N Neg** | **AUCROC** | **p-value** |
| --- | --- | --- | --- | --- | --- |
| **1: Sensitivity** | **Parent** | 30 | 270 | 0.723 (0.661, 0.786) | 0.012* |
|  | **Child** | 119 | 1071 | 0.912 (0.883, 0.938) | < 0.001* |
| **2a: Specificity** | **Parent** | 30 | 270 | 0.672 (0.611, 0.731) | < 0.001* |
|  | **Child** | 119 | 78 | 0.515 (0.433, 0.598) | < 0.001* |
| **2b: Strict Specificity** | **Parent** | NA | NA | NA | NA |
|  | **Child** | 105 | 78 | 0.442 (0.349, 0.532) | < 0.001* |

Table S12. AUCROC results for Brief Problem Monitor. P-values reported are two-sided. Significant p-values (<0.05) are marked with an asterisk (*).
